# Comprehensive analysis of normal adjacent to tumor transcriptomes

**DOI:** 10.1038/s41467-017-01027-z

**Published:** 2017-10-20

**Authors:** Dvir Aran, Roman Camarda, Justin Odegaard, Hyojung Paik, Boris Oskotsky, Gregor Krings, Andrei Goga, Marina Sirota, Atul J. Butte

**Affiliations:** 1 Institute for Computational Health Sciences, University of California, San Francisco, CA 94158 USA; 2 Department of Cell and Tissue Biology, University of California, San Francisco, CA 94143 USA; 3 Biomedical Sciences Graduate Program, University of California, San Francisco, CA 94143 USA; 4 Department of Pathology, Stanford University Medical Center, Stanford, CA 94305 USA; 5 Department of Pathology, University of California, San Francisco, CA 94143 USA; 6 Department of Medicine, University of California, San Francisco, CA 94143 USA; 7 Helen Diller Family Comprehensive Cancer Center, University of California, San Francisco, CA 94115 USA; 8 Korea Institute of Science and Technology Information, Biomedical HPC Research Center, 245 Daehak-ro, Yuseong-gu, Daejeon Korea

## Abstract

Histologically normal tissue adjacent to the tumor (NAT) is commonly used as a control in cancer studies. However, little is known about the transcriptomic profile of NAT, how it is influenced by the tumor, and how the profile compares with non-tumor-bearing tissues. Here, we integrate data from the Genotype-Tissue Expression project and The Cancer Genome Atlas to comprehensively analyze the transcriptomes of healthy, NAT, and tumor tissues in 6506 samples across eight tissues and corresponding tumor types. Our analysis shows that NAT presents a unique intermediate state between healthy and tumor. Differential gene expression and protein–protein interaction analyses reveal altered pathways shared among NATs across tissue types. We characterize a set of 18 genes that are specifically activated in NATs. By applying pathway and tissue composition analyses, we suggest a pan-cancer mechanism of pro-inflammatory signals from the tumor stimulates an inflammatory response in the adjacent endothelium.

## Abstract (web summary)

Normal tissue adjacent to the tumour (NAT) is often used as a control in cancer studies. Here, the authors analyse across cancer types the transcriptomes of healthy, NAT, and tumour tissues, and find that NAT presents a unique state, potentially due to inflammatory response of the NAT to the tumour tissue.

## Introduction

The regions immediately surrounding tumors have many morphologic and phenotypic distinctions from non-tumor-bearing healthy tissue, including pH levels^1^, allelic imbalance and telomere length^2^, stromal behavior^3^, and transcriptomic and epigenetic aberrations^4^. These substantial phenotypic and genetic changes are apparent up to 1 cm from the margins of the tumor. Therefore, histologically normal samples dissected adjacent to the tumor but beyond the observed aberrations (hereby referred to as NAT, normal adjacent to tumor), are frequently designated as healthy control samples for cancer studies under the assumption that histological normalcy implies biological normalcy. This approach has many advantages, such as allowing a comparison between samples from the same individual, often from a single larger tissue specimen, thus reducing individual-specific and anatomical site-specific effects. However, little is known about NAT tissue on the molecular level and whether it is truly “normal”. Perhaps, this is due to shortage of samples from non-diseased individuals, which are often difficult to obtain. Indeed, the limited number of studies that have characterized the NAT tissue relative to healthy tissues have focused on breast tissue, where healthy marginal tissue samples can be readily obtained from reduction mammoplasty and prophylactic mastectomy^5–7^.

The study of NAT tissue has been debated since Slaughter et al.^8^ first described the “field cancerization” theory, suggesting a cumulative process of carcinogenesis in which genetic alterations are acquired step-wise, leaving the NAT tissue in an intermediate, pre-neoplastic state composed of morphologically normal but molecularly altered cells. Recent studies on breast NAT suggested that the microenvironment surrounding the tumor, not the epithelial cells, is essential for understanding recurrence and in developing surgical strategies^9^. Moreover, NAT tissue gene expression is enriched for stromal pathways^10^, prominently consistent with wound response pathways^11^. Thorough evaluations have suggested that NAT tissue undergoes extracellular matrix remodeling, wound healing-like processes, fibrosis, and an epithelial-to-mesenchymal transition (EMT)^3^. Other studies focusing on prostate^12^, liver^13^, and colon^14^ have broadened the scope of NAT characterization; however, no multi-tissue multi-cancer evaluation has been performed to date, and a full characterization of NAT tissue is lacking.

The Genotype-Tissue Expression (GTEx) program^15^ is a multicenter effort to generate genomic and transcriptomic profiling data for >50 tissue sites from hundreds of autopsies. The Cancer Genome Atlas (TCGA)^16^ is another multicenter effort to produce molecular profiling data from thousands of cancer patients across >30 cancer types. In ~10% of these samples, the TCGA program also generated molecular profiling data of NAT tissues. According to TCGA protocols, NAT samples must be collected >2 cm from the tumor margin and/or must not contain tumor by histopathologic review^17–22^. By combining the data from GTEx and TCGA, we broaden the scope of NAT characterization from studies focusing on single tissue types to a more systematic analysis of eight distinct tissues and their corresponding tumors (together referred to as tissue types), representing the most common solid malignancies. Although there are many differences between tissue types, we focused this study on the shared elements of NAT across tissue types, which have not been evaluated to date. This expanded analysis allowed us to interrogate general mechanisms by which tumors interact with its surrounding tissue. We performed a comprehensive analysis of transcriptomic profiles from healthy tissue, NAT, and tumor, including dimensionality reduction, differential expression, protein–protein interactions (PPI), gene-set enrichment, and tissue composition analyses to provide a coherent picture of NAT tissue characteristics. Our analyses showed that the NAT tissue is distinct from both healthy tissue and tumor and represents an intermediate state between them. We uncovered NAT-specific characteristics, namely activation of pro-inflammatory immediate-early response genes concordant with endothelial cell stimulation. We suggest that the induction of this NAT-specific signature is orchestrated by the tumor, spreading pro-inflammatory signals to its surroundings. Our cross-tissue analysis allowed us to detect pan-cancer characteristics, and we suggest that stromal changes in NAT represent an emerging hallmark of cancer that may be essential for tumorigenesis and/or tumor progression.

## Results

### Integrative analysis of TCGA and GTEx RNA-seq data

**Fig. 1:**
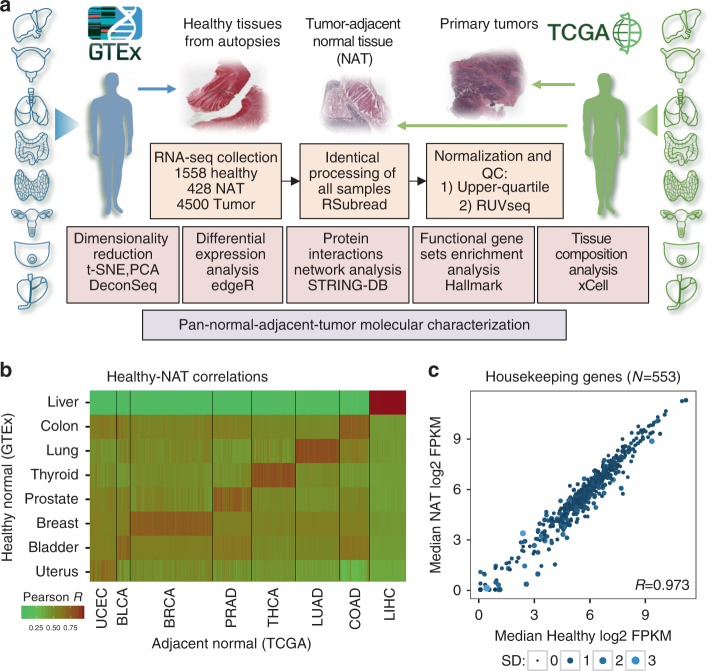
Comparison of healthy tissues, normal, adjacent normal (NAT) tissues, and tumors. **a** Study design. From GTEx, we collected 1578 RNA-seq raw samples across bladder, breast, colon, liver, lung, prostate, thyroid, and uterus tissues, and matched with corresponding tumor types 428 normal adjacent tumor (NAT) and 4500 tumor samples from TCGA. We performed identical processing of all samples using the protocol presented in Rahman et al.^23^, and validated that the data are coherent. We then utilized several techniques to characterize the differences between healthy tissues, NAT, and tumor tissues that are shares across tissue types. Credit for the organs illustrations in this figure: © Alex Oakenman/Shutterstock.com. All rights reserved. These images are not included under the creative commons license for this article. **b** Pearson correlation between median healthy samples in each tissue site (rows) and each of the 428 NAT samples. In 405 of the NAT samples (94.6%), the maximal correlation coefficient was with the corresponding healthy tissue. **c** Median log2 expression levels of 553 housekeeping genes in healthy and NAT tissues across tissue types. Spearman coefficient is presented. The size of the point represents the standard deviation (SD) in NAT, and color represents SD in healthy. High concordance in SD is observed between NAT and healthy as well (*R* = 0.902)

An identical analysis pipeline is required to allow rigorous comparison of the transcriptomic profiles from TCGA and GTEx (Fig. 1a). To this end, we obtained raw RNA-seq reads of GTEx samples and applied the exact pipeline used for the production of raw counts of mapped reads previously described^23^. We combined these with TCGA raw count data analyzed using the same pipeline and compiled a transcriptomic dataset comprising of 1558 healthy normal samples, 428 NAT samples, and 4500 primary tumor samples across eight tissue types (Table 1). Note that although GTEx tissue donors cannot be referred to as “healthy” as they comprise a broad range of non-cancer disease processes, which led to death^24^, we designate them as such in this study to connote that none of the donors were diagnosed with cancer. The collective cancer patients were significantly older than the patients contributing the healthy samples, 12.2 years on average, in most tissues. The only exception is thyroid, where the cancer patients tended to be younger than those individuals from which the healthy samples were obtained.

**Table 1:** Number of samples and demographics of samples included in the study

GTEx	TCGA	# of samples			Sex (% of females)			Age (mean ± SD)		
		H (healthy normal)	A (NAT)	T (tumor)	H	A	T	H	A	T
Lung	LUAD	374	59	541	34.2	56.1	53.1	52.1 ± 12.0	66.0 ± 11.0	65.9 ± 9.8
Colon	COAD	376	41	483	40.0	53.8	48.0	50.4 ± 12.6	70.7 ± 13.5	67.6 ± 13.1
Breast	BRCA	92	113	1119	100	100	100	51 ± 11.5	58.0 ± 14.4	59.1 ± 13.1
Uterus	UCEC	90	35	554	100	100	100	47.5 ± 13.4	59.9 ± 12.1	64.4 ± 11.1
Liver	LIHC	135	50	374	32.6	42.9	32.5	52.7 ± 11.3	61.3 ± 16.2	59.8 ± 13.4
Bladder	BLCA	11	19	414	46.2	47.4	26.4	42.5 ± 14.1	70.4 ± 11.3	68.6 ± 10.6
Prostate	PRAD	119	52	502	0.0	0.0	0.0	49.0 ± 13.6	60.9 ± 7.1	61.3 ± 6.8
Thyroid	THCA	361	59	513	36.5	70.7	72.9	52.0 ± 11.9	46.2 ± 17.2	47.8 ± 15.8

*H* healthy normal from GTEx; *A* normal adjacent tumor (NAT) samples from TCGA; *T* tumor samples from TCGA

To concordantly analyze expression profiles from TCGA and GTEx, we first verified comparability between the two datasets. To do so, we started by analyzing fragments per kilobase of transcript per million (FPKM) values and correlating median expression profiles of the eight healthy tissue types with all NAT samples. Altogether, 94.6% of the NAT samples were correctly correlated with the corresponding healthy tissue (Fig. 1b), supporting general comparability between the datasets. Another way to test for differential batch effects is by comparing the expression and variation of housekeeping genes. We correlated the median expression levels of housekeeping genes^25^ across all non-tumor samples and found a strikingly high degree of agreement between the datasets (Pearson *R* = 0.973, *p-*value < 1 × 10^−20^) (Fig. 1c; Supplementary Fig. 1). Moreover, we observed a high level of agreement when comparing the variation of expression levels within the dataset (Spearman *R* = 0.902, *p-*value < 1 × 10^−20^) (Supplementary Fig. 1). Although we admit a perfect study would involve simultaneously obtained freshly collected normal and cancer samples from the same individuals, these findings demonstrate that GTEx and TCGA can be analyzed jointly.

### NAT tissue is distinct from both healthy and tumor tissues

**Fig. 2:**
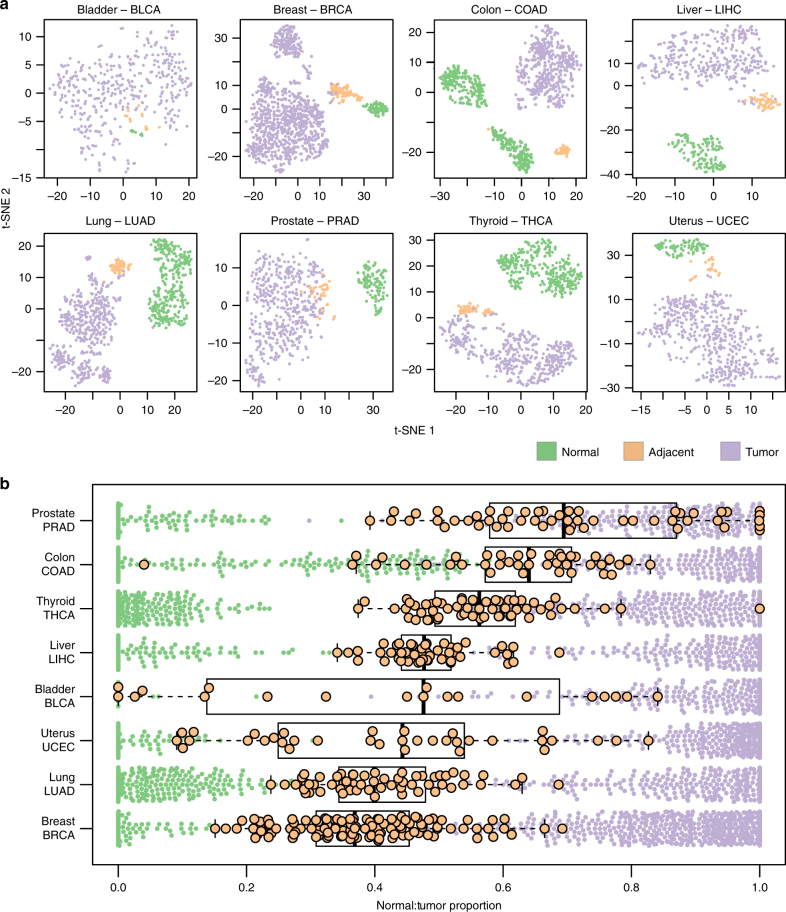
Intermediate state of NAT between healthy and tumor tissue. **a** t-SNE plots for each tissue types. Each group is clustered on its own in all plots. In 6 of 8 plots, the NAT samples (orange) are in between the healthy (green) and tumor (purple) samples. In bladder, there is not sufficient power of non-tumor samples compared with the tumor obstructing the discrimination between the conditions, yet the NAT samples are in between the tumor and the healthy tissue. Colon is an exception because of an issue related to the source of the healthy samples. **b** Deconvolution analysis of the NAT samples using median expression levels of healthy and tumor as references. The result of the analysis is the fraction of similarity of each NAT sample to the tumor. The small points, normal (green) and tumor (purple) deconvolution fractions, are shown as reference

We performed dimensionality reduction for each tissue type’s transcriptome. In these analyses, we used counts per million (CPM) values normalized with the upper quartile normalization method, excluding genes with low read abundance due to the pronounced differences in library sizes between TCGA and GTEx (Supplementary Fig. 2). Strikingly, the same trend was observed in all tissue types: the three conditions were clearly distinguished, with NAT samples found between tumor and healthy samples (Fig. 2a; Supplementary Fig. 3). Thus, across disparate tissue contexts, NAT is a distinct tissue type that reproducibly segregates between healthy and tumor, as predicted by the field cancerization theory, and may not be appropriately categorized as “normal”.

The disparities between the source datasets for healthy and NAT tissues above represent a major potential weakness of this analysis. Thus, to validate our findings we searched public data repositories for smaller independent studies that collected samples from all three conditions jointly. Our search yielded four microarray cohorts with sufficient sample sizes in colon^14^, liver^13^, breast^26^, and prostate^27^. Using comparison methodology similar to that described above, healthy, NAT, and tumor tissues cleanly segregated as seen in the initial comparisons in the colon, liver, and breast cohorts (Supplementary Fig. 4); a trend towards this pattern of segregation was also observed in the prostate cohort (Supplementary Fig. 5). Interestingly, NAT segregated into a transcriptional state intermediate between healthy and normal in the colon, liver, and prostate cohorts; however, NAT from the independent breast cohort did not, which could be explained by the differences in array designs between healthy and NAT/tumor.

Another concern is the imbalanced group sizes; however, a reduced analysis with an equal number of samples per group reiterated our findings that NAT displays an intermediate expression state between healthy and tumor (Supplementary Fig. 6). Finally, an independent reanalysis of the TCGA and GTEx samples using the Toil pipeline^28^ reaffirmed our findings as well, confirming the uniqueness of NAT is not a result of improper analysis (Supplementary Fig. 7).

The dimensionality reduction analyses suggest that there are differences between tissue types, such that the expression profiles of NAT are closer to the tumor cluster in some tissues and closer to the healthy tissue cluster in others. To better quantify this phenomenon, we employed a deconvolution pipeline^29^, to calculate a “normal:tumor” fraction for all samples. Our analysis revealed substantial differences among NATs from different tissue types (Fig. 2b). Expression profiles of NAT from breast, colon, liver, lung, and uterine tumors—all malignancies that tend to produce tumors with grossly and histologically well-defined borders—cluster distinctly from those of both normal and tumor. On the other hand, prostate NAT samples—a tumor that seamlessly infiltrates surrounding “normal” tissue, often without forming discrete tumor-normal boundaries—is highly similar to a portion of the tumor profile, suggesting perhaps microscopic contamination of NAT samples with tumor and tumor samples with NAT. This same phenomenon may also be seen in certain—but not all—types of thyroid cancer, which could possibly explain the intermediate (partly overlapping, partly distinct) nature of this dataset as well.

In colon, we observed two healthy clusters (Fig. 2a). Deeper analysis revealed that these clusters are from different sections of the colon, sigmoid, and transverse, and suggested that the closer resemblance of NAT to the tumor (Fig. 2b) is due to heterogeneity in the GTEx dataset. Indeed, subsequent analyses bifurcated the healthy-NAT relationship by anatomic site (Supplementary Fig. 8), with NAT from the sigmoid colon more closely resembling the tumor, whereas NAT from the transverse colon more closely resembles healthy tissue.

### Shared gene signatures differ NAT from healthy tissue

**Fig. 3:**
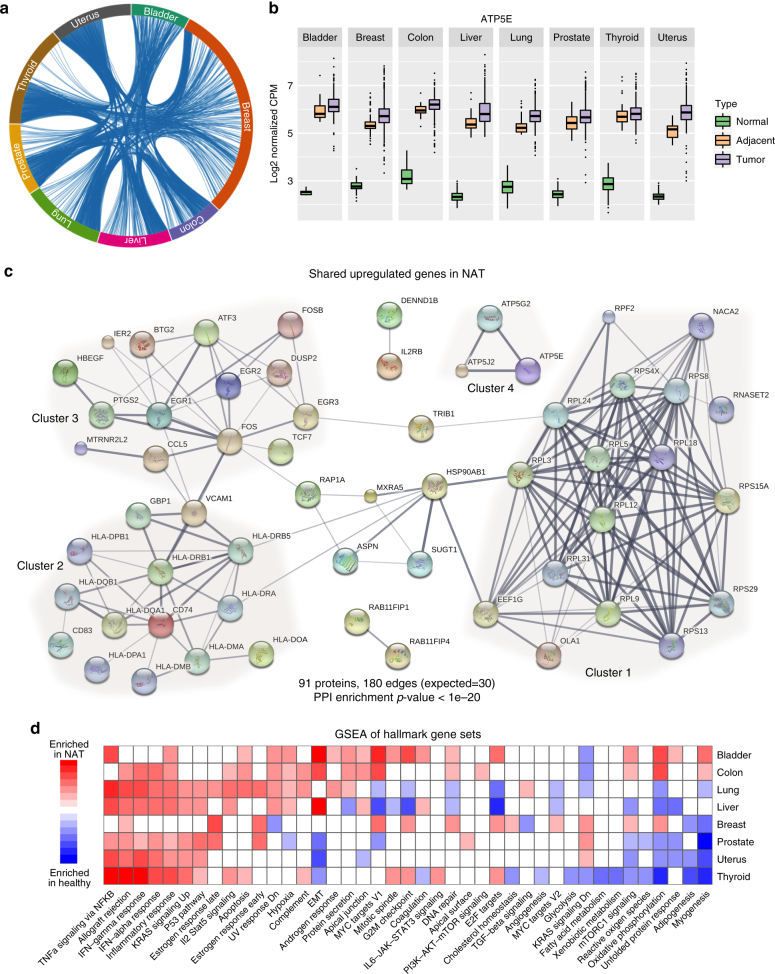
Upregulated genes in NAT compared with healthy. **a** Overall, 2451 genes were upregulated in NAT compared with healthy across all tissue types. Of those, 660 were found in more than one tissue site, 223 in more than two (*x*-fold more than expected by random) and 98 in more than three (*x*-fold). The chord diagram shows the vast amount of shared genes among all tissue types. **b** Boxplot of the expression levels of ATP5E, an example of a gene that is consistently upregulated in NAT compared with healthy. No significant difference is observed between NAT and tumor. **c** STRING analysis of protein–protein interactions of the 98 genes, corresponding to 91 proteins, upregulated in NAT compared with healthy in at least four tissue types. A total of 180 edges are found between 57 of the genes (other genes not shown). Only 30 are expected by chance (Poisson approximation *p*-value < 1 × 10^−20^). Thickness of edges indicates confidence. We observed four clusters with three or more genes cluster 1: cell division; cluster 2: immune response; cluster 3: cellular stimuli; cluster 4: ATP. **d** Gene-set enrichment analysis (GSEA) of the hallmark gene sets using NAT vs. healthy differential expression. NES are presented, but only for significant comparisons (FDR < 1%). Otherwise, the color of the cell is white. Only gene sets significant in at least one tissue site are presented. The full data is in Supplementary Data 3. Inflammatory response-related pathways are generally enriched in NAT in most tissue types (red). On the other hand, the NAT tissue tends to not express normal development pathways such as myogenesis and adipogenesis (blue)

To further explore the details that define this divergence between healthy tissue and NAT, we performed differential expression analyses across tissue types. To remove possible confounding differences in sample preparation and batch effects that may occur because of the different data sources, we used stringent removal of unwanted variation^30^, diminishing the variations between datasets (Supplementary Fig. 9). Yet, we identified on average 440 upregulated and 554 downregulated differentially expressed genes (DEGs) in NAT relative to healthy tissues across all tissue types (Supplementary Data 1; Supplementary Table 1). Remarkably, we found widespread similarities in upregulated and downregulated DEGs across tissue types (Fig. 3a). We identified 98 genes that are upregulated in at least four tissue types (80-fold more than expected by random, Poisson approximation *p-*value < 1 × 10^−20^), and 164 genes as downregulated (47-fold, Poisson approximation *p-*value < 1 × 10^−20^) (Supplementary Data 2). Overall, 61.2% of the upregulated genes and 73.2% of the downregulated genes showed a tendency for upregulation or downregulation across all tissue types (Fig. 3b; Supplementary Figs. 10 and 11). PPI analysis revealed a remarkably significant enrichment of known interactions among those genes (STRING PPI enrichment *p-*value < 1 × 10^−20^) (Fig. 3c). Four strongly connected components shared across tissue types were clearly observed: a ribosomal biogenesis component (cluster 1) and several genes involved in oxidative metabolism (cluster 4) indicating high transcriptional activity in the NAT compared with healthy tissue; an immune component (cluster 2), enriched for antigen-processing genes, suggesting increased activation of the immune response; and a component enriched for immediate-early genes (cluster 3), associated with a wide variety of cellular stimuli and known to be widely induced during stress responses^31^. Interestingly, no interactions or pathway enrichment were observed in the 164-shared downregulated DEGs in NAT (Supplementary Fig. 12), emphasizing the uniqueness of the shared upregulated genes.

To gain further insight into the global patterns that distinguish between healthy tissue and NAT, we performed a gene-set enrichment analysis (GSEA) using the 50 hallmark gene sets^32^. Altogether, 41% of all comparisons between healthy tissue and NAT were significantly perturbed (GSEA nominal *p*-value false rate discovery (FDR) < 1%) (Fig. 3d; Supplementary Data 3). Examining the trends of divergence across tissue types revealed robust enrichment of inflammatory response-related gene sets upregulated in NAT, such as tumor necrosis factor (TNF)-α signaling, interferon response, and allograft rejection. Interestingly, several cancer-related signatures were also enriched in NAT, such as *KRAS* signaling, p53 pathway, hypoxia, and apoptosis. On the other extreme, cellular differentiation and metabolic pathways, such as myogenesis, adipogenesis, and oxidative phosphorylation, respectively, were significantly enriched in most healthy tissues.

### Characterizing a general gene expression profile for NAT

**Fig. 4:**
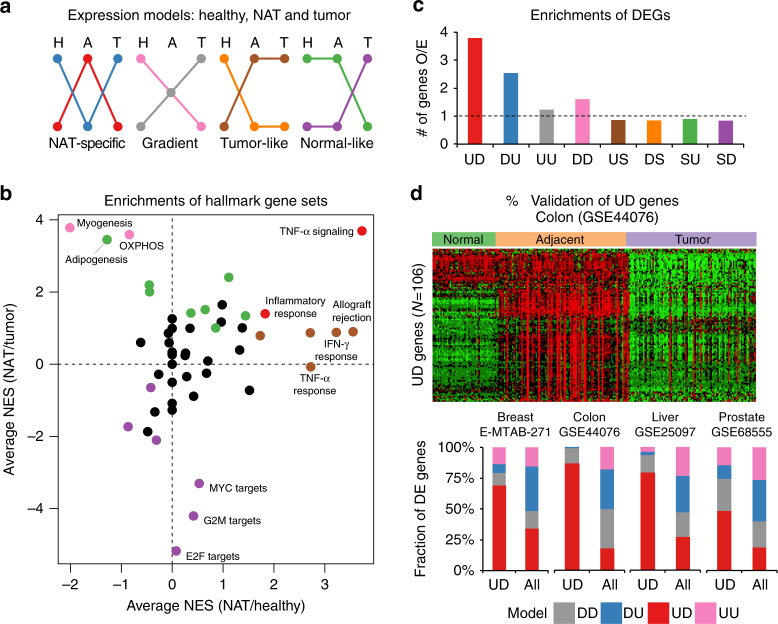
NAT expression compared with healthy and tumor. **a** Genes and gene sets expression profiles were divided to nine expression models: each gene/gene set can be upregulated (U), downregulated (D) or not differentially expressed (stable, S) in NAT vs. healthy and tumor vs. NAT. Expression models suggest a NAT-specific activation or repression (UD/DU models), an intermediate state between normal and tumor (UU/DD), resembling healthy (SU/SD) or resembling tumor (US/UD). The null model (SS) is not presented. **b** Normalized gene-set enrichment score (NES) of hallmark gene sets in NAT compared with healthy (*x* axis) and compared with the tumor (*y* axis). Non-significant NES values (FDR < 1%) were nullified. Gene sets were colored according to the expression models in (**a**) if they fit the expression model in the majority of tissue sites. NES are positive if enrichmed in NAT. Cancer-related pathways (bottom), correspond to the SD model; inflammatory-related pathways (right), correspond to the US model; normal development gene sets (top-left), correspond to the DD model; the TNF-α signaling pathway has a NAT-specific UD activation model. **c** Average fold change of the number of observed genes in each expression model in each tissue site compared with the expected number of genes by the number DEGs in A:H and in T:H. The NAT-specific UD and DU models are highly enriched compared with null hypothesis. **d** Validation of UD genes in independent cohorts containing healthy, adjacent, and tumor samples. Top: heatmap of gene expression in colon (GSE44076), where 106 of 119 UD genes are found. In 92 of the genes, the average expression in NAT is higher than in healthy and tumors. Bottom: in four microarray cohorts we classified our tissue type identified UD genes to four categories: higher average in NAT compared with both healthy and tumor (UD); lower average in NAT (DU); average in NAT lower than healthy but higher than tumor (DD); higher average in NAT than healthy but lower than tumor (UU). In all four cohorts, the UD model was highly enriched compared to expected (colon: 86.8% (expected–17.7%); liver: 79.2% (27%); breast: 69% (34.1%); Prostate: 48.4% (18.8%))

We further computed differential expression patterns between NAT and tumor samples (Supplementary Data 4) and divided all DEGs between healthy, NAT, and tumors into nine models of expression change: upregulation/downregulation/stable between healthy and NAT (A:H—Adjacent:Healthy) and between NAT and tumor (T:A—Tumor:Adjacent) (Fig. 4a). Analyzing the aforementioned hallmark sets we found that 55.8% of the comparisons between NAT and tumors showed significant differentiation (FDR < 1%), and 30.5% were significant in both NAT vs. healthy and NAT vs. tumor (NAT-specific or gradient models) (Supplementary Fig. 13; Supplementary Data 3). In general, cancer-related gene sets, such as MYC and E2F targets and G2M, showed a “normal-like” tendency in NAT; normal cellular differentiation pathways, such as adipogenesis and myogenesis, showed a “gradient” tendency; and inflammatory-related pathways showed a “tumor-like” tendency (Fig. 4b). One gene set, the TNF-α signaling pathway, strikingly presented a “NAT-specific” activation in seven of the examined tissues.

We next analyzed individual differentially expressed genes. Remarkably, of the nine models described above, only NAT-specific upregulation or downregulation models were enriched compared with a null hypothesis (Fig. 4c; Supplementary Table 2). On average across tissue types, we identified 82 genes that were upregulated compared with both healthy tissue and tumor (TASA, tumor-adjacent specific activation), 3.8-fold more than expected by the upregulated in A:H and downregulated in T:A in each tissue type. This result suggests that NAT tissue represents not just a gradient between tumor and healthy tissue or contamination resulted by infiltrating tumor cells, but is instead a distinct tissue phenotype. Examining the four independent microarray cohorts strongly validated the NAT-specific activation of the identified TASA genes (Fig. 4d; Supplementary Figs. 14–17). Gene ontology (GO) analysis revealed a strong enrichment of extracellular matrix (ECM)-associated genes within TASA genes in seven out of eight tissue types (Supplementary Fig. 18), in accordance with previous findings of ECM enrichment in the tissue surrounding the tumors^11^.

**Fig. 5:**
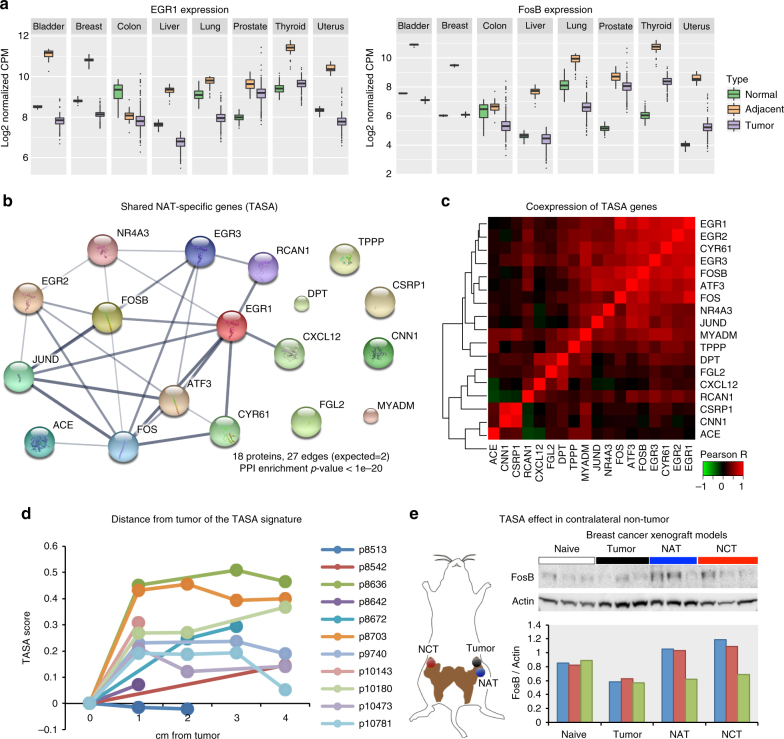
Shared tumor-adjacent normal (NAT)-specific genes. **a** Boxplot of the expression levels of EGR1 and FOSB, examples of gene that are upregulated in NAT compared with healthy and downregulated in tumors in most tissue types. **b** STRING analysis of protein–protein interactions (PPI) of 18 genes specifically activated in NAT in at least three tissue types. Overall, 27 edges are found between 12 of the genes. Only 2 are expected by chance (PPI enrichment *p*-value < 1 × 10^−20^). Thickness of edges indicates confidence. **c** Co-expression analysis of the 18-shared TASA genes in NAT samples. Same as in the PPI analysis, we observe strong co-expression of 12 of the genes, but in addition, we observe that more associations between genes on top of the PPI analysis. **d** TASA scores (ssGSEA of 18-shared TASA genes) in 11 breast tumors patients and in the adjacent tissue, up to 4 cm from the tumor boundaries (E-TABM-276 study). In each patient, scores are aligned relative to the tumor. In 10 of 11 patients, an increase in TASA score was observed outside of the tumor. The TASA score increases immediately outside the tumor (1 cm) and is maintained across the adjacent tissue. In 4 of the 6 patients with multiple expression profiles, we observed a small decrease in TASA score in 4 cm compared to 1 or 2 cm, possibly suggesting a modest gradient effect as a function of the distance from the tumor. **e** Top: western blot analysis of FosB protein levels in tumor, NAT and contralateral non-tumor (NCT) mammary gland of three human breast cancer patient-derived xenografts (HCI-002, HCI-009, and HCI-010). In addition, 3 naive mouse mammary glands are shown as reference. Bottom: FosB levels normalized to actin levels. Blue = HCI-002, red = HCI-009, and green = HCI-010. In two of the PDXs (HCI-002 and HCI-009), we observed a marked elevation of FosB levels in both the NAT and NCT compared with the samples from the tumor and non-tumor from naive mice

Prominent TASA genes include *EGR1/2* and *FOS/FOSB* (Fig. 5a). Previous studies have shown downregulation of the mRNA of these genes in different cancer types compared to NAT^33, 34^. A study in pancreatic cancer showed higher levels of *c-FOS* protein in NAT compared with the pancreatic tumors^35^. Importantly, our results suggest that these genes are not, in fact, downregulated in the tumor itself but rather they are specifically activated in NAT, in contrast to previous studies. Altogether, we identified 18 TASA genes shared by at least three tissues (Supplementary Figs. 19 and 20). Strikingly, 12 of these genes comprised a PPI network (PPI enrichment *p-*value < 1 × 10^−20^) (Fig. 5b). Co-expression analysis revealed tight co-expression across tissue types between most of the TASA genes (Fig. 5c).

### Upstream regulators of the NAT-specific gene signatures

Intriguingly, TASA genes are highly enriched with immediate-early response genes (seven genes), a gene family that is rapidly and transiently upregulated following external stimuli such as growth factors, hormones, or stress^31^. Thus, we explored whether the tumor itself may be the source of these external stimuli. According to this hypothesis, the TASA signature should decrease as a function of the distance from the tumor. To this end, we analyzed a unique dataset, which examined the transcriptomes of multiple regions surrounding breast tumors^36^. In 10 of 11 patients, a TASA score, calculated using single-sample gene-set enrichment analysis (ssGSEA)^37^ for the 18-shared TASA genes, was enriched in the adjacent regions, providing a validation that this score does indeed identify NAT (Wilcoxon rank test *p-*value = 0.002). Remarkably, when applied to this spatial dataset, the TASA score remained elevated compared with the tumor even 4 cm from the tumor, suggesting that primary tumors can exert influence over a substantial distance (Fig. 5d). In four of six patients with multiple samples, we observed a modest decrease 4 cm from the tumor. Although the sample number is small, this tendency for decrease of TASA score as a function of distance might suggest a gradient effect, which remains to be examined in more samples and more distant regions.

The long distance activation of the TASA signature in NAT suggested a systemic effect. To test whether the TASA signature is elevated away from the tumor, we utilized a panel of human breast cancer patient-derived xenograft (PDX) mouse models^38^, and measured the relative protein expression level of FosB, a prominent TASA gene, whose expression is highly correlated with most of the TASA signature. In two out of the three PDXs, FosB was markedly elevated in NAT compared with both the tumor and non-tumor mammary gland from naive mice. Moreover, this elevation was further observed in the contralateral non-tumor mammary gland (NCT) (Fig. 5e). Although the close proximity effect observed in NAT can be explained by proximal stress response, it cannot explain the effect observed in NCT. In addition, PDX models do not appear to undergo a de novo tumor evolutionary process and are also immunocompromised^38^, thus rejecting the field effect hypothesis and immune response-related explanations. Therefore, a more appropriate explanation suggests that the distance-driven effect is intermediated by putative signals secreted by the tumor, which may exercise influence far away from tumor margins.

We therefore examined possible growth factors and upstream regulators that can be secreted by the tumor and activate this signature. Twelve of the TASA genes (66.7%) were upregulated in response to TNF-α, as suggested by pathway analysis, but other cytokines, growth factors, and chemical compounds have also been shown to activate this network (Supplementary Fig. 21; Supplementary Data 5). According to a literature-curated database^39^, TNF was suggested as an upstream regulator for genes specifically activated in NAT as well as in the non-shared genes in six of the eight tissues (Supplementary Table 3). Other attractive candidates are platelet-derived growth factor (PDGF)-BB, which has a significant role in blood vessel formation, and leukotriene D, a lipid-based inflammatory mediator that increases vascular permeability.

We also attempted to identify putative regulators of the TASA signature empirically. Thus, we correlated the TASA scores in 415-matched NAT and tumor samples across tissue types, sorted by the correlation coefficient, and investigated the highly ranked genes (Supplementary Data 6). Among the top ranked genes across tumor types, we detected several potential regulators for the TASA signature, including SERPINE1 and IL6, which are known pro-angiogenic factors^40^. HB-EGF, Heparin-binding EGF-like growth factor, was found in the top 1% of half of the tumor types. This protein has a significant role in the development of malignant phenotypes by contributing to metastasis and invasion by promoting EMT and angiogenesis^41^. Our data here suggest it is also involved in reshaping the adjacent tissue, possibly further enabling tumor progression.

### NAT cellular composition

We previously had shown that cellular composition has a profound effect on the construction of co-expression networks^42^. Previous studies have shown induction of these genes in endothelial cells, fibroblasts, and other stromal cells^43–45^, thus we hypothesized that TASA signatures are specifically activated in tissue stroma. Using our recently developed method xCell^46^, which employs a compendium of gene signatures with a removal of dependencies between closely related cell types, we estimated the enrichment of 30 immune and stromal cell types that reside in tumors and their adjacent tissues (Supplementary Data 7).

The analysis revealed major differences in the abundance of many cell types not just between the tissue types, but also between healthy, NAT and tumors (Supplementary Figs. 22 and 23). Interestingly, a t-SNE plot of all samples based on the cell types profiles revealed that tumors across tissue types cluster together (excluding liver). In contrast, non-tumor samples tend to cluster according to the tissue type, such that there is a cluster for each tissue type that includes both the healthy and the NAT samples (Supplementary Fig. 24). Thus, while the cellular composition of normal tissues is stable, irrespective of its proximity to a tumor, the microenvironment inside the tumor is strongly perturbed from its tissue of origin and has similar tendencies across tumor types.

**Fig. 6:**
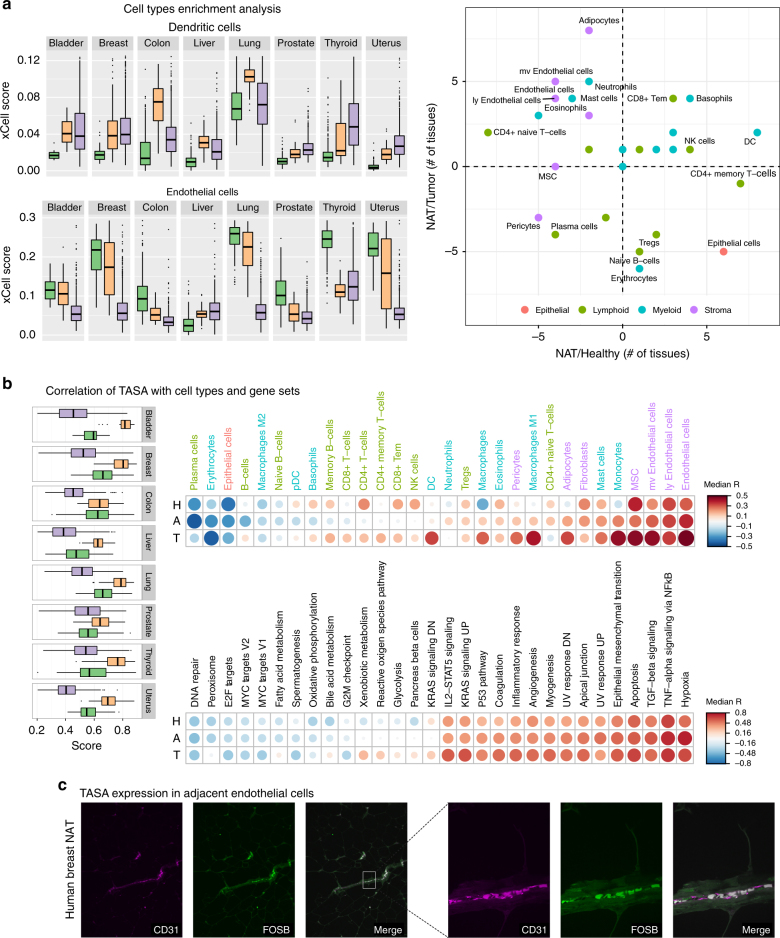
Cell types and pathway analysis of the NAT-specific activation signature. **a** Left: boxplot of the xCell scores for dendritic cells (DC) and endothelial cells (EC). DCs tend to be low in healthy samples and higher in NAT and tumors. ECs are high in normal samples, tend to be lower in NAT, and even lower in tumors. Right: Scatter plot of the differential number of tissue types where the cell type is significantly enriched between NAT and healthy (*x* axis), and NAT and tumor (*y* axis). For example, endothelial cells are significantly diminished in five NAT tissues compared with healthy (breast, colon, lung, and thyroid) and enriched in one tissue type (liver)—thus the *x* value is −4. Significance analysis was performed using Mann–Whitney test, and a significant difference was defined as Bonferroni corrected *p*-value < 0.001. **b** Left: Boxplots of ssGSEA scores of the 18-shared TASA signature. In 7 of 8 tissue types, there is significant enrichment in NAT compared with both healthy and tumor. In colon, there is no enrichment compared with healthy, and can be explained by the differential expression of the TASA genes between sigmoid and transverse colon (Supplementary Fig. 26). Top: median Spearman coefficients across tissue types between TASA scores and xCell scores. Cell types were ordered according to the NAT coefficients. Top correlations are with endothelial cells, suggesting their role in these cells. Down: median Spearman coefficients across tissue types between TASA scores and hallmark gene sets. Gene sets were ordered according to the NAT coefficients. Only top and bottom 15 genes sets are presented. TASA is positively correlated with pathways that induce epithelial–mesenchymal transition. **c** Immunofluorescent staining for CD31, an endothelial cell marker, and FosB protein in NAT of a human breast tumor excision specimen (two other samples are in Supplementary Fig. 29). Remarkably, both markers are highly colocalized in all three samples (Costes *p*-value < 1 × 10^−6^)

Across tissue types we observed several cell types with a tendency to differentiate NAT from healthy or tumors (Fig. 6a). Dendritic cells are highly enriched in NAT compared with healthy tissues across all tissue types, but are less divergent between NAT and tumors. Cell types that are classically recruited in inflammatory response, including memory CD4+ T-cells, NK cells, and basophils, tended to be enriched in NAT compared to healthy, congruent with our previous findings of increased expression of inflammatory markers in NAT compared to healthy tissue (Supplementary Figs. 22 and 23). On the other hand, endothelial cells showed a tendency to be depleted in NAT compared to healthy tissue, but compared to tumor show an even stronger tendency to be enriched. Interestingly, previous studies have suggested that NAT tissue is enriched with endothelial cells^10^; however, our analysis suggests that they are not actually enriched in NAT but are rather strongly depleted in the tumor itself, with NAT representing an intermediate state (Supplementary Fig. 22). In summary, our analysis suggests that inflammation, a cancer hallmark, is also strongly present in the adjacent tissue, whereas endothelial cells are somewhat less prevalent in NAT compared with the healthy and even less well-represented in the tumor in a gradient-like fashion.

We next calculated a TASA score, based on the 18-shared TASA genes, and found that it is highly enriched in NAT in most tissue types, with relatively low variation between NAT samples (Fig. 6b). This enrichment was further validated in the independent microarray datasets (Supplementary Fig. 25). In colon, the TASA score did not differ between healthy and NAT in our TCGA-GTEx dataset, but did differ in the independent dataset, probably attributed again to the aggregation of divergent profiles from the sigmoid and transverse sections (Supplementary Fig. 26). Correlating TASA scores with cell type scores revealed that it is robustly associated primarily with endothelial cells, but also with mesenchymal stem cells (MSC), adipocytes, monocytes, and mast cells (Fig. 6b; Supplementary Fig. 27). In tumors, but less so in NAT and not at all in healthy, the TASA signature also strongly correlated with activated macrophages, which are a primary source of TNF-α^47^. This result might be related to our suggestion above that the tumor is the source of the factors that induce the TASA signature in NAT. We also correlated TASA scores with ssGSEA scores of hallmark gene sets (Supplementary Fig. 28). Here, we observed high concordance between TASA and hallmark gene sets across tissue types, regardless of tissue condition, revealing a high prevalence of gene sets representing hypoxia, TNF-α and TGF-β signaling, apoptosis, EMT, and angiogenesis (Fig. 6b), in accordance with our previous findings of enrichment of these pathways in NAT.

To test our gene expression-based association between endothelial cells and the TASA score, we collected excision specimens from three human breast tumors with clear adjacent regions. By staining for CD31 (an endothelial cell marker) and FosB in the NAT region, we saw a remarkable co-localization of these markers (Fig. 6c; Supplementary Fig. 29). We conclude that the TASA signature is highly activated specifically in the NAT endothelial cells.

### NAT can control for differential expression in cancer studies

**Fig. 7:**
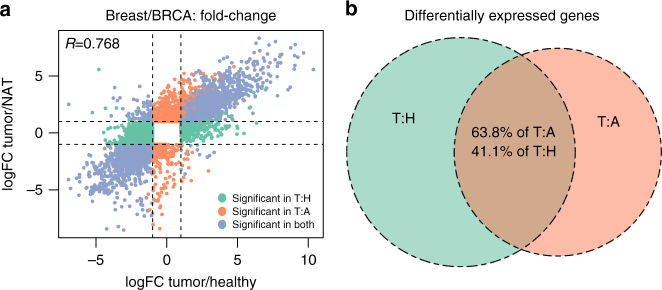
Comparison of differential expression analysis with healthy tissue or NAT as controls. **a** Scatter plot of log_2_ fold-changes in differential expression analyses between tumor and healthy (*x* axis) or NAT (*y* axis) as control. Pearson coefficient is presented. **b** Venn diagram of differentially expressed genes (DEGs) in tumor vs. healthy (T:H) and tumor vs. NAT (T:A) across all tissue types. 63.8% of T:A DEGs are also significant in T:H, 41.1% of T:H DEGs are also significant in T:A

The analyses presented above highlight a crucial consideration when performing differential expression analysis in cancer research: the control set used in the analysis has a substantial impact on findings. To demonstrate this issue we compared the results from DEG analysis of tumor vs. NAT (T:A) and tumor vs. healthy normal (T:H). The overall Pearson correlation of the fold-changes between the analyses ranged 0.416–0.768 (Fig. 7; Table 2; Supplementary Fig. 30). We found 55.3% more significant DEGs in tumor vs. healthy (T:H) than in tumor vs. NAT (T:A). Across all tissues, 63.8% of the significant DEGs in T:A are also significant in T:H (Fig. 7b). Finally, only a very limited number of the significant DEGs are discordant between analyses: 58 genes are upregulated in T:A and downregulated in T:H on average, and 46 show the opposite discordance (Fig. 7c). Thus, we conclude that although using NAT as a control adequately identifies the majority of differentially expressed genes in tumors, using healthy tissues is more accurate and provides additional information obscured when using NAT.

**Table 2:** Differential expression analysis of tumors vs. healthy tissue or NAT as controls

Tissue	Tumor type	*R*(logFC)	T:H DEGs	T:A DEGs	DEGs in both	Up in T:H, down in T:A	Down in T:H, up in T:A
Bladder	BLCA	0.687	2804	1909	1163	2	2
Breast	BRCA	0.768	6523	4500	3408	10	83
Colon	COAD	0.650	5978	4345	2420	90	63
Liver	LIHC	0.632	4291	3286	1852	14	135
Lung	LUAD	0.673	4982	4727	2704	69	46
Prostate	PRAD	0.416	5606	2076	1164	32	91
Thyroid	THCA	0.491	4028	2245	1204	114	36
Uterus	UCEC	0.762	8190	4217	3507	36	10

T:H—tumor vs. healthy. T:A—tumor vs. NAT. *R*(logFC)—Pearson coefficient between log fold-changes of T:H and T:A. The T:H/T:A DEGs columns present the number of significant genes found in each analysis. “DEGs in both” is the intersection of both analyses. The last two columns show discrepancies in the called direction of change from each analysis

## Discussion

It is clear that we must understand the myriad ways in which cancer cells interact with their immediate, local, and remote environments if we are to understand how tumors form and thrive. This understanding, in turn, is vital for effective prevention and therapy. In the last decade, several studies have investigated gene expression profiles of tumors’ surroundings, but the biological significance of these findings remains poorly understood.

The study of tumor biology, irrespective of approach, requires controls. Using normal adjacent tissue as this control has many advantages, such as the relative ease of access and the control for variability between individuals and anatomic sites; however, in comparing only tumor and NAT tissues, many potential cancer biomarker candidates may be missed and others spuriously implicated. Moreover, given the critical importance of NAT in tumorigenesis, this approach may also obscure therapeutics targets present within the NAT itself. Here, we show that understanding the molecular differences between tumor-adjacent and healthy tissues can directly reveal mechanisms used by the tumor to communicate with the surrounding tissue.

The power of this study stems from its multi-tissue, multi-cancer approach; by broadening the experimental aperture from a single tissue and/or single tumor type to integrating information across tissues types, we uncovered important general attributes of tumor-adjacent tissues. It is clear that different cancer types present unique tissue characterizations, such as the fibrosis or cirrhosis of the liver in most of the hepatocellular carcinoma patients; however, our findings suggest that a universal mechanism that characterizes NAT is apparent as well. Here, we have shown that NAT is distinct from both healthy and tumor tissues, and that many of these differences are not unique to a particular tumor type but tend to be shared across types. We identified a set of genes that are specifically overexpressed in NAT tissues compared with both healthy tissues and tumors, and demonstrated a strong association between this signature and TNF-α and TGF-β signaling pathways, hypoxia, and EMT. It is important to emphasize that studies that do not include healthy tissues will misidentify TASA genes as selectively under-expressed in the tumor, whereas in reality, these are actually expressed at normal levels in the tumor but selectively induced in surrounding tissue.

Several cancer development theories can explain this NAT-specific activation. Cancer often arises in the context of prolonged inflammation^48^. The TASA signature, we uncovered may be involved in systemic inflammation and specifically induced in the acute phase reaction. However, according to this hypothesis, it would be expected that this signature be also activated in tumors, not specifically in NAT as we observed. The “wound that never heals” theory^49^, which implies that cellular and biochemical processes associated with wound healing are similar to those involved in the growth and development of tumor stroma, strongly coincides with our findings of TASA signature activation and the enrichment of inducers of blood vessels. However, this theory concerns tumor stroma and not tumor-adjacent tissue, which is histologically normal.

Here we explored a mechanism of tumor–environment interaction in which tumor-secreted factors influence the surrounding tissue to promote tumor invasion and metastasis. Tumor hypoxia, for example, is responsible for the expression of many different factors that induce EMT and vessel formation. One such factor is *HB-EGF*, whose expression in tumors is associated with activation of the TASA signature in paired NAT tissue^50^. We suggest that secretion of this and other factors by the tumor activates a cascade of transcription factors and enzymes associated with the induction of TNF-α and TGF-β signaling pathways, which, in turn, are prominent inducers of EMT, and are strongly activated in the adjacent endothelium. In contrast to the field cancerization theory, which implies an evolutionary process that forms the NAT phenotype prior to frank tumorigenesis, we argue that the tumor itself has an active role in shaping a unique, dynamic phenotype in its adjacent tissue. Strong support for this notion comes from our PDX experiment, where field effect or immune response cannot explain the FosB activation in the contralateral mammary gland. We suggest that the interaction with the tumor may help shape the adjacent microenvironment, assembling its unique tissue composition and inducing signaling pathways responsible for the formation of the tissue.

A major limitation inherent to the integration of multiple independently collected datasets is disparity between sample sets. In this study, NAT and healthy tissues came from different projects with different sample collection and sequencing protocols. We have attempted to remedy this by standardizing analysis pipelines, employing contemporary methods for removal of unwanted variation, and confirming our findings in multiple datasets generated by orthogonal methodology; however, we cannot entirely disprove the possibility of batch effects. As with any computationally based study, independent confirmation is necessary for any further conclusions to be drawn. The simple experiments we performed are just a first step towards such experimental validation, and demonstrate that it is possible to study NATs in tumor models such as PDXs. Another limitation of this study is that no clear distance of the NAT samples is available; thus, we were unable to determine whether the observed NAT characteristics are unique to proximal non-tumor cells or part of the disease process of entire organs that have been exposed to carcinogenic stress. More data, such as the breast cancer study, we re-analyzed^36^, containing multiple samples around the tumor, could facilitate a better understanding of the tumor surroundings and evaluation of our hypotheses.

Although the mechanisms that alter gene expression in NAT remains to be validated, it is clear that NAT tissue has unique characteristics differentiating it from healthy tissues. The strong NAT-shared components across tumor types and tissue types suggest that this editing of the adjacent tissue—more specifically, the adjacent stroma—is an important mechanism, possibly orchestrated by the tumor itself. Pietras and Östman^51^ suggested that the interactions with the tumor stroma should be considered as a hallmark of cancer. Here we broaden the scope of this hallmark to include the adjacent stroma as well. We also suggest that disruption of this complex interplay might represent a potential novel therapeutic strategy in the treatment of cancer.

## Methods

### Data collection and processing

The analysis in this study focused on eight tissue types and tumor types, which contain a sufficient number of NAT samples in TCGA (*N* > 10), and the tissue of origin of the tumor is clear. For the eight tumor types, we analyzed raw feature counts and FPKM values were downloaded from NCBI’s Gene Expression Omnibus (GEO) via accession number GSE62944^23^. We further obtained raw reads files of the eight corresponding tissue types (GTEx dbGaP accession phs000424.v6.p1, 18 November, 2015). The raw reads were then processed and normalized using the Rsubread package (version 1.14.2)^52^ and aligned to the UCSC hg19 reference genome according to the pipeline described in Rahman et al.^23^ The summary of the number of samples is presented in Table 1, and the processed GTEx expression profiles were deposited to GEO (accession number GSE86354). We also obtained counts per million (CPM) values following upper quartile normalization using the EDASeq package^53^.

Other datasets included in this study were downloaded from GEO (GSE44076, GSE25097, GSE16113, GSE68555, and GSE5364) or EMBL-EBI ArrayExpress (E-TABM-276). Raw CEL files were downloaded and processed using custom CDFs from BrainArray (GSE44076, GSE68555, and E-TABM-276)^54^ and GEO (GSE25097). The processing and normalization were performed using the Robust Multi-array Average (RMA) procedure on Affymetrix microarray data. GSE16113 was not reprocessed, as it was not analyzed by a standard microarray.

### Data analysis and statistical methods

Dimensionality reduction. Dimensionality reduction was performed using the Rtsne (version 0.10) package and the EDASeq package on the log_2_ CPM values (RNA-seq), or log_2_ RMA values (microarray). The deconvolution procedure was performed using the DeconRNASeq package^29^. This algorithm adopts a globally optimized non-negative decomposition algorithm through quadratic programming for estimating the mixing proportions of distinctive tissue types. Here we used two distinct tissue types: the average expression levels of the healthy samples and the tumor samples. Thus, the result of this procedure is a proportion of the “tumor contribution” to the sample. Only genes with at least 10 reads in at least two samples were included for the analysis.

Differential expression analysis. Batch effects and differences in sample preparation can have substantial ramifications on the outcomes. Thus, we performed a recently published stringent removal of unwanted variation method for RNA-seq. We employed the RUVg method from the RUVSeq package^30^, which performs factor analysis on residuals using a negative gene set that has constant covariates. The negative set we used was a list of housekeeping genes^55^, which were suggested by the developers of the method. This procedure diminished the variations between datasets, as can be observed in the relative log expression (RLE), which were typically low (<1) and undistinguishable between the conditions (Supplementary Fig. 9). The normalization procedure was performed between pairs of the three conditions in each tissue site independently, and differential expression analysis was then performed using edgeR^56^ (Supplementary Datas 1 and 4). Only genes with at least 10 reads in at least two samples were included for the analysis. A gene was considered as differentially expressed if (1) Bonferroni corrected *p*-value < 0.05, (2)>2-fold expression change, and (3) log_2_ CPM > 3. Genes were divided to the nine expression models using the same rules—a gene with an upregulation in NAT compared with healthy and downregulation in tumor compared with NAT will be assigned to the “UD” expression model (or TASA) in Fig. 4a and b (in red).

The chord diagram was created using NetworkAnalyst website (http://www.networkanalyst.ca/). PPI networks were created using the STRING website (http://string-db.org/). Upstream regulators analysis was performed using the Ingenuity Pathway Analysis software.

Statistical significance test. To calculate *p*-values for the observed shared number of genes across tissues in the different analyses, we used Poisson approximation of the Binomial distribution for a null hypothesis of independence between the tissues^57^. PPI enrichment *p*-values are presented as reported by the STRING webtool.

Gene-set enrichment and tissue composition analyses. The GSEAPreranked software^58^ was used to calculate normalized enrichment scores (NES) and (FDR) values for the 50 Hallmark gene sets^32^. The genes were preranked according to the log fold-change values. NES corresponds to the enrichment score (ES), which reflects the degree to which a gene set is overrepresented at the top or bottom of a ranked list of genes. The normalization is based on the gene-set enrichment scores for all dataset permutations.

Tissue composition analysis of 30 immune and stroma cell types, those that are assumed to reside in the tumor and tissue microenvironment, was performed using xCell (version 1.0)^46^. xCell is a gene signatures-based method, which employs a compensation technique to reduce spill-over effects between closely related cell types. ssGSEA implemented in the GSVA package^59^ was used to score samples according to the FPKM expression values of the 18-shared TASA genes.

### Orthotopic xenograft studies

PDX samples (HCI-002, HCI-009 and HCI-010) were generated and published by Dr Alana Welm and colleagues at the University of Utah following local institutional review and patient consent. Briefly, donated primary breast tumors and metastatic breast cancer cells were freshly obtained following surgery and transplanted into cleared mammary fat pads of female immunocompromised NOD/SCID mice^38^. For this study, we obtained 4-week-old immunocompromised NOD/SCID/gamma female mice purchased from Jackson Laboratory. The viably frozen HCI-002, HCI-009, and HCI-010 tumor samples were transplanted into the cleared inguinal 4R mammary fat pads of NOD/SCID/gamma mice. Tumor growth was monitored daily by caliper measurement in two dimensions. When tumors reached 2 cm in any dimension (HCI-002—after 8 weeks on average, HCI-009 and HCI-010—12 weeks), mice were killed, and tumor and NAT isolated from the 4R gland, and NCT from the 4L gland, and flash-frozen in liquid nitrogen. The protocols described in this section regarding animal studies were approved by the UCSF Institutional Animal Care and Use Committee.

### Immunoblot analysis

Proteins were extracted using RIPA buffer (50 mM Tris-HCl pH 7.6, 150 mM NaCl, 0.5% sodium deoxycholate, 1% Triton X-100, 0.1% SDS, 2 mM EDTA) and proteinase (Roche) plus phosphatase (Roche) inhibitor cocktails. Protein extracts were resolved using 4–12% SDS-PAGE gels (Life Technologies) and transferred to nitrocellulose membranes (Life Technologies). Membranes were probed with primary antibodies overnight on a 4 °C shaker, then incubated with horseradish peroxidase (HRP)-conjugated secondary antibodies, and signals were visualized with ECL (Bio-Rad). The primary antibodies targeting the following proteins were used: β-actin (actin) (sc-47778 HRP, Santa Cruz, 1:10,000) and FosB (2251, Cell Signaling, 1:1000).

### Immunofluorescence staining and microscopy

Breast cancers used for immunofluorescence were identified and retrieved from the clinical archives of the University of California San Francisco (UCSF) Department of Pathology. All tumors consisted of estrogen receptor (ER)-positive, progesterone receptor (PR)-positive, HER2-negative invasive ductal carcinomas. Breast tissue was fixed in 10% formalin and embedded in paraffin. Tumor blocks with sufficient tumor and adjacent (at least 0.5 cm) normal tissue were selected, and 4 µm sections were cut on plus-charged slides for immunofluorescence. This study was approved by the UCSF institutional review board. For immunofluorescence labeling, slides were dewaxed in xylene followed by rehydration in graded ethanol (100, 95, 70%) and deionized H_2_O. Antigen retrieval was performed in 10 mM Tris, 1 mM EDTA, 0.05% Tween 20, pH 9 at 121 °C for 4 min. Subsequently, tissue sections were blocked in 1% bovine serum albumin, 2% fetal bovine serum in PBS for 5 min, and incubated with primary antibodies (CD31, 3528, Cell Signaling, 1:100 and FosB, 2251, Cell Signaling, 1:100) overnight at 4 °C. Following several PBS washes, sections were incubated with Alexa Fluor-488 or -568 conjugated antibodies, counterstained with DAPI (Sigma), and mounted using Vectashield (Vector). Epifluorescence images were acquired by spinning disk microscopy on a customized microscope setup as previously described^60–62^ except that the system was upgraded with a next generation scientific CCD camera (cMyo, 293 Photometrics) with 4.5 μm pixels allowing optimal spatial sampling using a ×60 NA 1.49 objective (CFI 294 APO TIRF; Nikon).

### Data availability

Processed GTEx expression profiles were deposited as GEO accession number GSE86354. All other datasets used in this manuscript are available in public repositories and references are given in the text (see “Data collection and processing” subsection).

## Electronic supplementary material

Supplementary Information

Description of Additional Supplementary Files

Supplementary Data 1

Supplementary Data 2

Supplementary Data 3

Supplementary Data 4

Supplementary Data 5

Supplementary Data 6

Supplementary Data 7
