# Supplementary material for: Comprehensive analysis of normal adjacent to tumor transcriptomes
Source: Nat Commun. 2017 Oct 20;8:1077. doi: 10.1038/s41467-017-01027-z (PMC5651823; doi:10.1038/s41467-017-01027-z)
Supplement: Supplementary file 1 — Supplementary Information [file 41467_2017_1027_MOESM1_ESM.pdf]

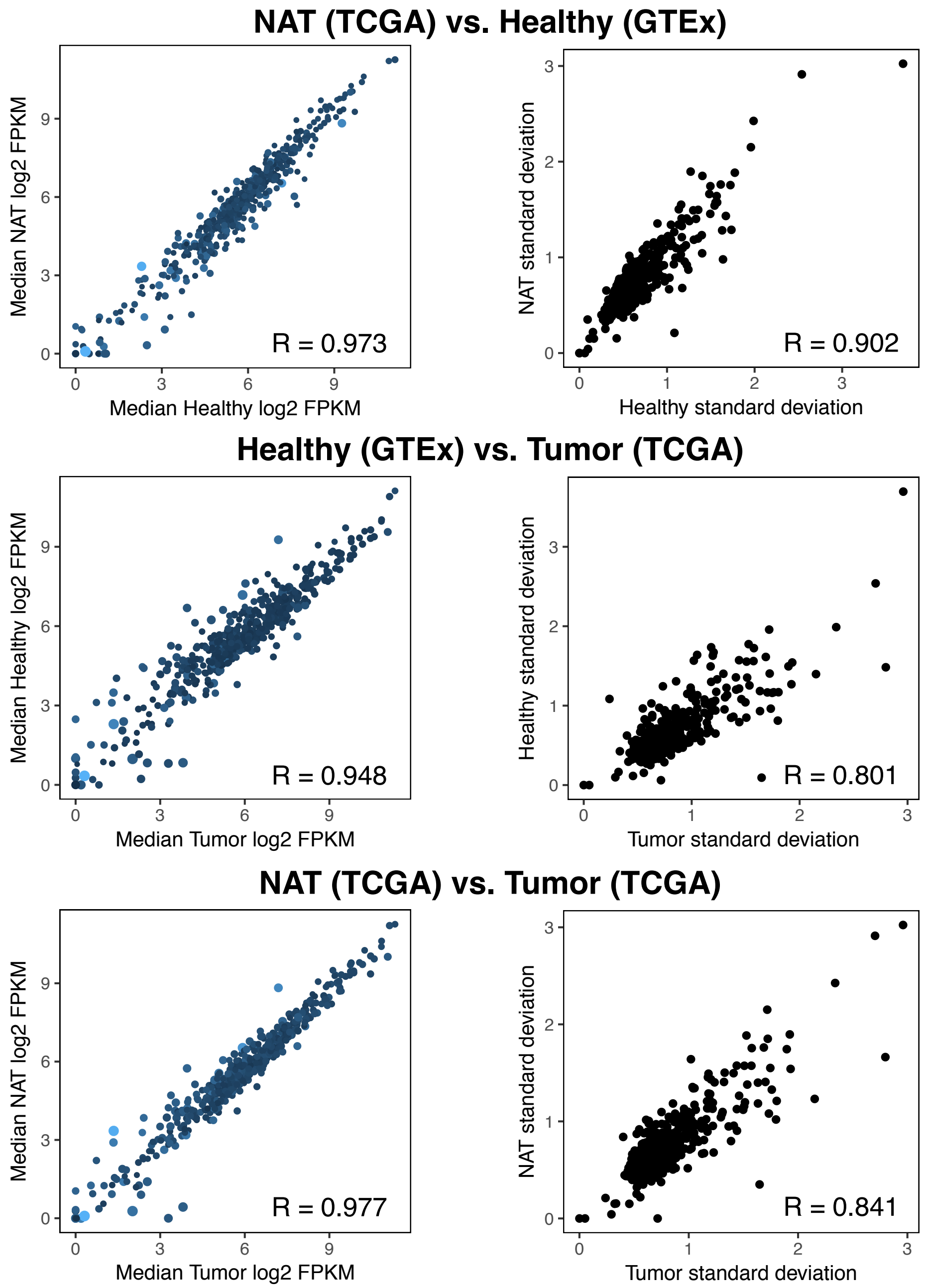

Supplementary Figure 1. Housekeeping genes variation between GTEx and TCGA. Scatter plots of the median expression levels (left) and standard deviation (SD) (right) of 553 housekeeping genes in 1,578 healthy samples from the GTEx project, 428 NAT samples and 4,500 tumor samples from the TCGA project. Pearson coefficient is presented. The standard-deviation correlation between non-tumors is higher than when comparing non-tumor with tumor, regardless of data source. The lower correlation can be explained by perturbation in house-keeping genes in tumors.

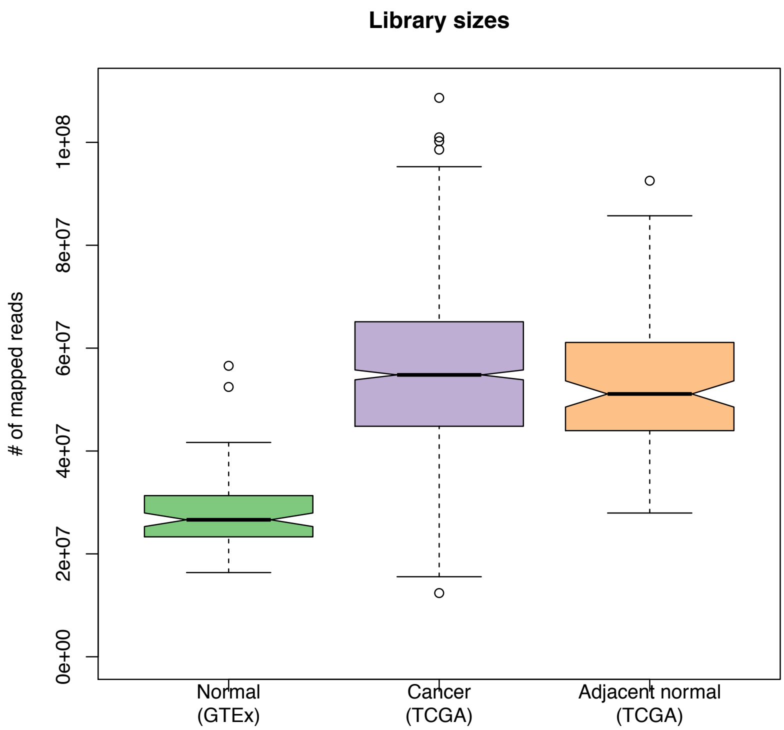

		Healthy (GTEx)	Tumor (TCGA)	Adjacent Normal (TCGA)
**Bladder**	BLCA	23,436,564	42,013,957	37,882,127
**Breast**	BRCA	26,630,340	54,798,411	51,091,841
**Colon**	COAD	25,151,349	33,902,081	44,219,371
**Liver**	LIHC	24,475,752	46,620,891	50,963,556
**Lung**	LUAD	28,736,898	41,067,778	33,296,387
**Prostate**	PRAD	24,402,067	46,901,636	55,611,732
**Thyroid**	THCA	26,624,138	57,384,211	58,618,181
**Uterus**	UCEC	28,018,125	23,212,459	39,693,535

Supplementary Figure 2. RNA-seq library size differences between GTEx and TCGA. Top: Box plot of the RNA-seq library sizes in the GTEx and TCGA samples. **Bottom:** Median library size of each tissue site.

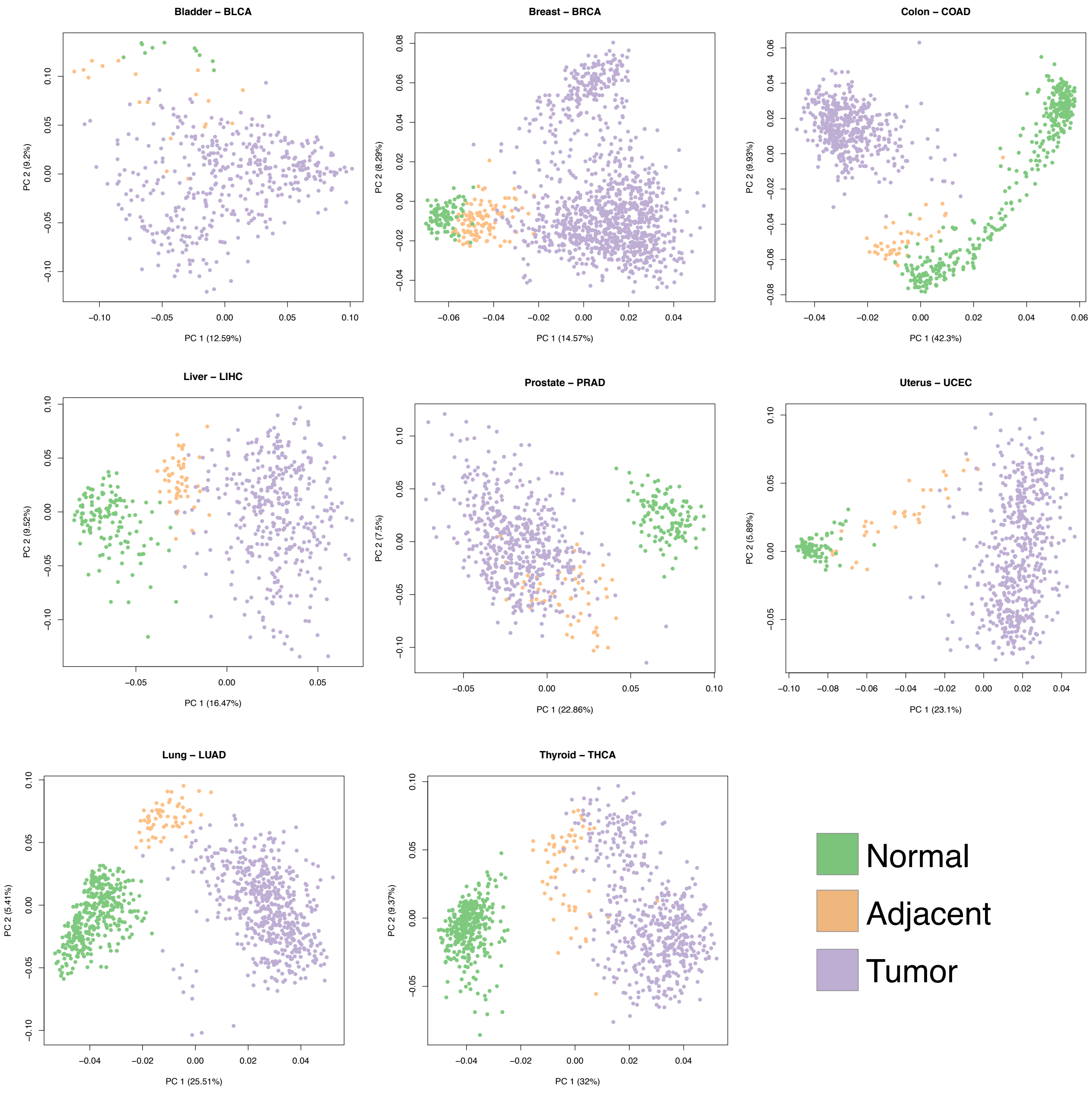

Supplementary Figure 3. PCA plots for healthy, NAT and tumor. Using CPM values we produce PCA plots for each of the tissue types. In all tissue types, except bladder were the number of non-tumor samples is low, each condition forms its own cluster. Moreover, in all of the NAT tissue is between the healthy tissue and the tumor clusters.

A

		# of samples		
**GEO dataset**	**Tissue type**	**Healthy**	**NAT**	**Tumor**
**GSE44076**	**Colon**	50	98	98
**GSE25097***	**Liver**	6	243	268
**GSE68555****	**Prostate**	65	182	195
**E-MTAB-271**	**Breast**	56	34	32

* Additional 40 cirrhotic samples.
** Divided in three different microarray platforms.

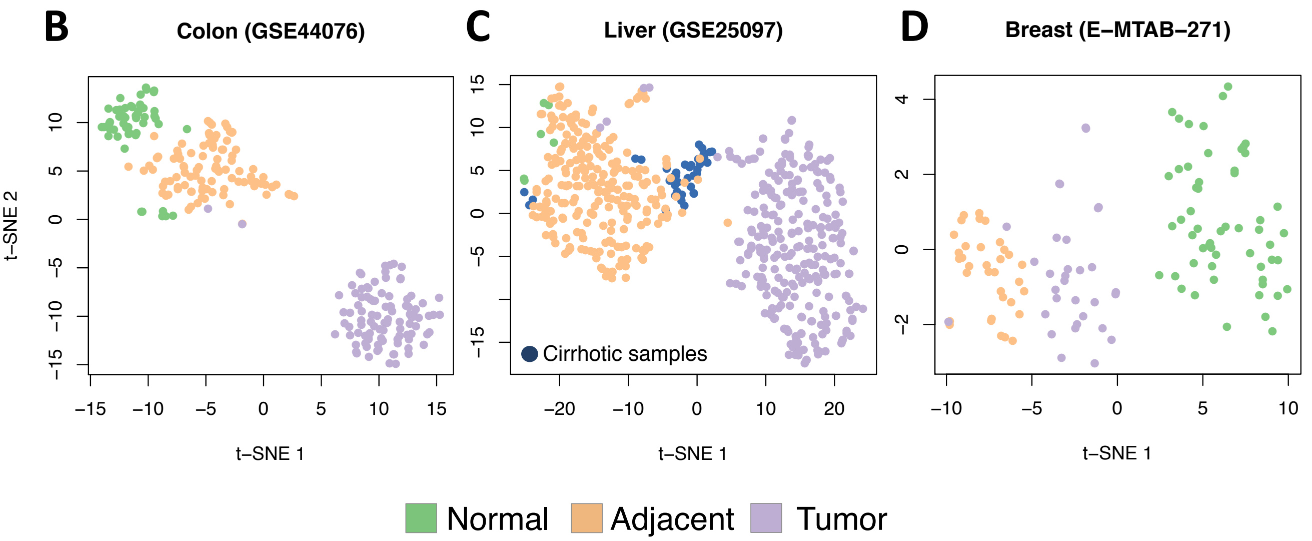

Supplementary Figure 4. Dimensionality reduction in independent cohorts – colon, liver and breast. t-SNE plots of microarray cohorts. **A.** Summary table of the samples used in each of the cohorts. **B.** GSE44076 contains paired NAT mucosa and tumor samples and healthy colon mucosa, analyzed using Affymetrix Human Genome U219 arrays. **C.** GSE25097 contains hepatocellular carcinoma tumors, adjacent non-tumors, cirrhotic and healthy liver samples, analyzed usingRosetta/Merck Human RSTA Affymetrix 1.0 arrays. The cirrhotic samples (blue) are intersecting the NAT samples but tend towards the tumor. **D.** E-MTAB-271 contains reduction mammoplasty from healthy donors, and malignant tumors and NAT samples from breast cancer patients, analyzed using Agilent Whole Genome Microarrays. The healthy samples were analyzed using Agilent Whole Human Genome Microarray 4x44K 014850 G4112F, while the non-healthy samples were analyzed using Agilent Whole Human Genome Oligo Microarray 012391 G4112A.

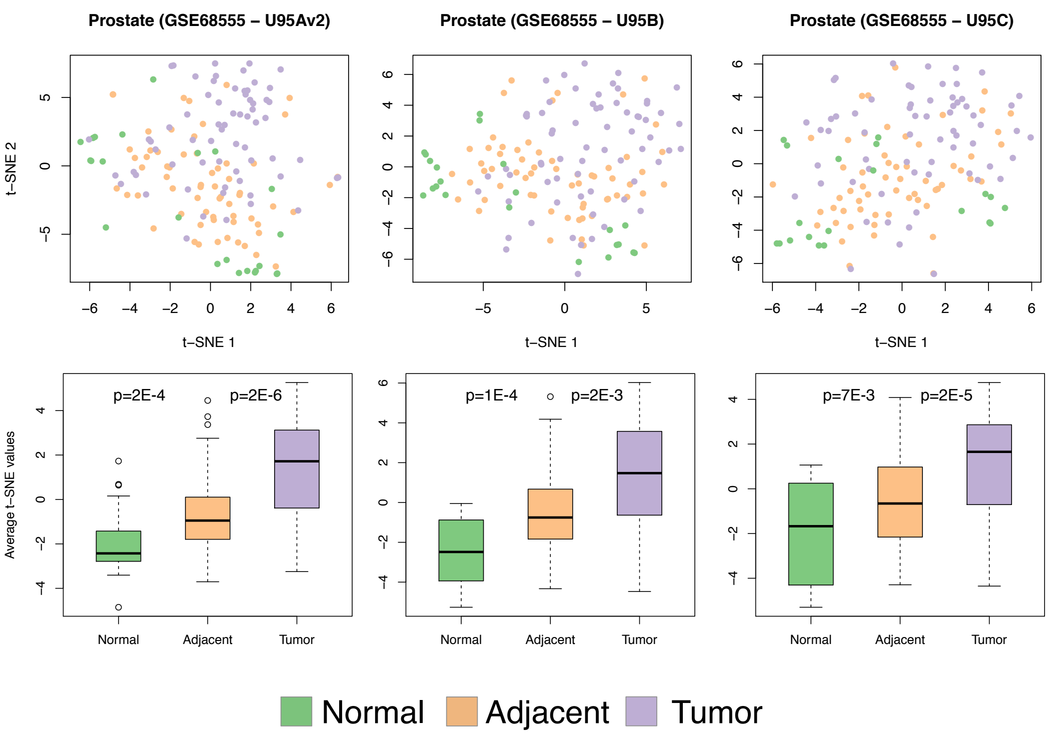

Supplementary Figure 5. Dimensionality reduction in independent cohorts - prostate. GSE68555 contains prostate cancer, NAT prostate, and organ donor prostate tissues. In this dataset there was no clear segregation between the conditions, supposedly because the samples were analyzed in a previous generation microarray platforms (Affymetrix Human Genome U95 Version 2 Array, U95B and U95C). Nevertheless, the box plots on the bottom, which present the average t-SNE values show that the NAT tissue is in intermediary state in the samples analyzed on the three platforms. Wilcoxon ranked-sum test p-values between sets are presented.

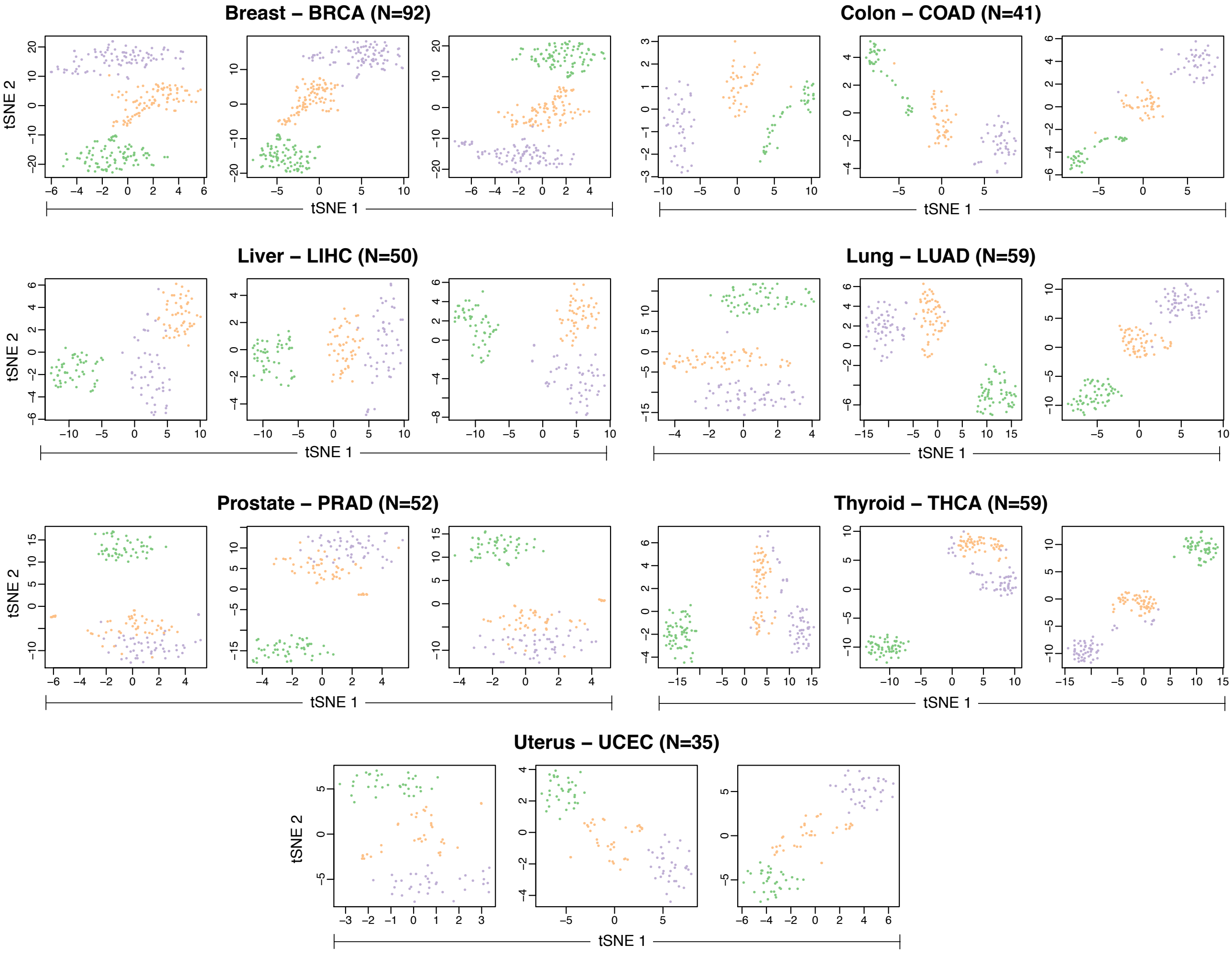

Supplementary Figure 6. Dimensionality reduction with equal sample sizes. Each row shows three t-SNE plots that were performed in the same manner as figure 2A, but with randomly selecting equal number of samples from each of the conditions. The analysis shows that the divergence between conditions we observe is not related to the low number of NAT samples compared to tumor and healthy.

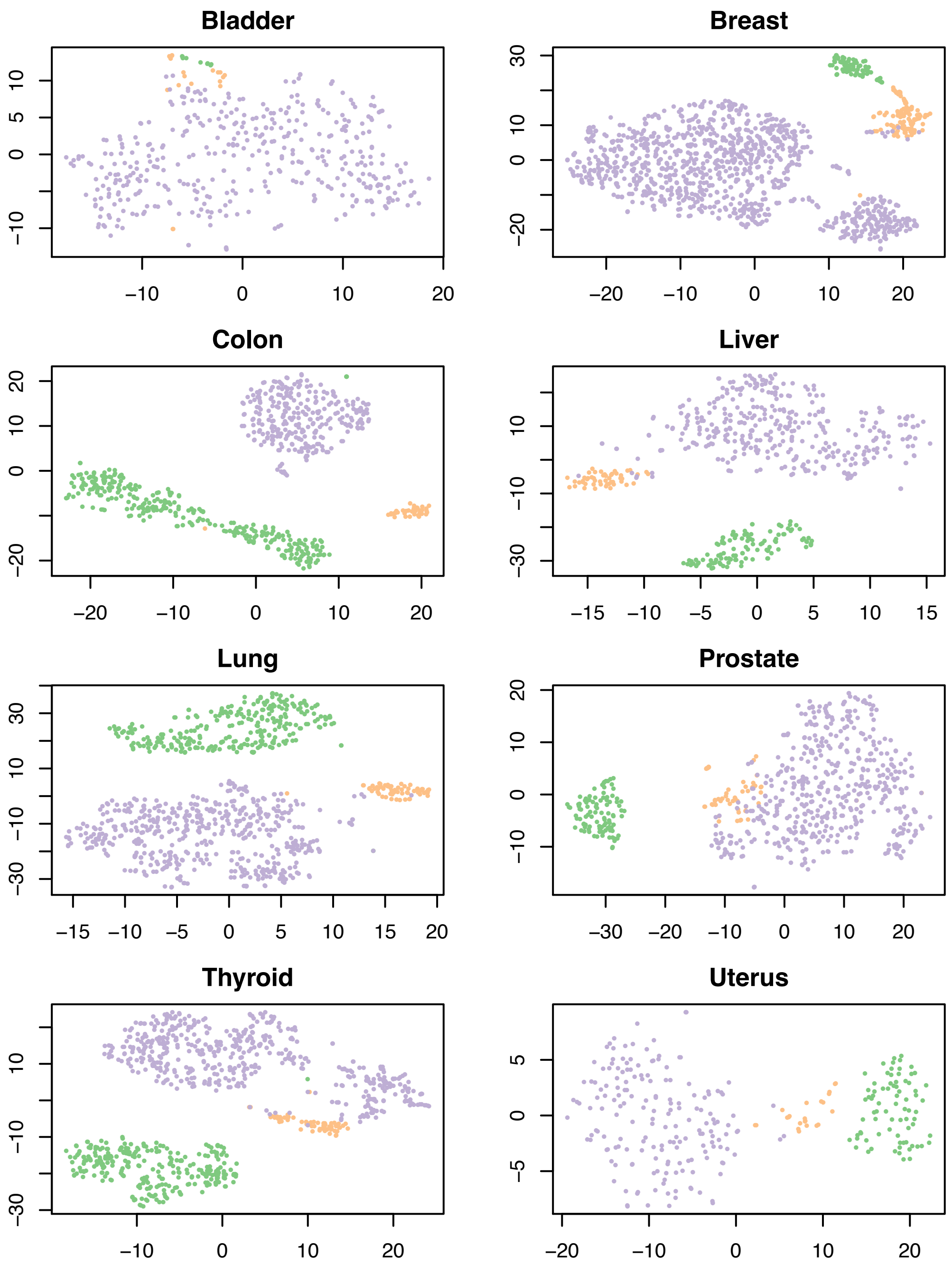

Supplementary Figure 7. Dimensionality reduction with Toil reanalysis. Vivian et al. (bioRxiv, 2016) reanalyzed all TCGA and GTEx samples using a workflow named Toil. We show that are result is not a byproduct of our reanalysis, but is also observed using other reanalysis workflows.

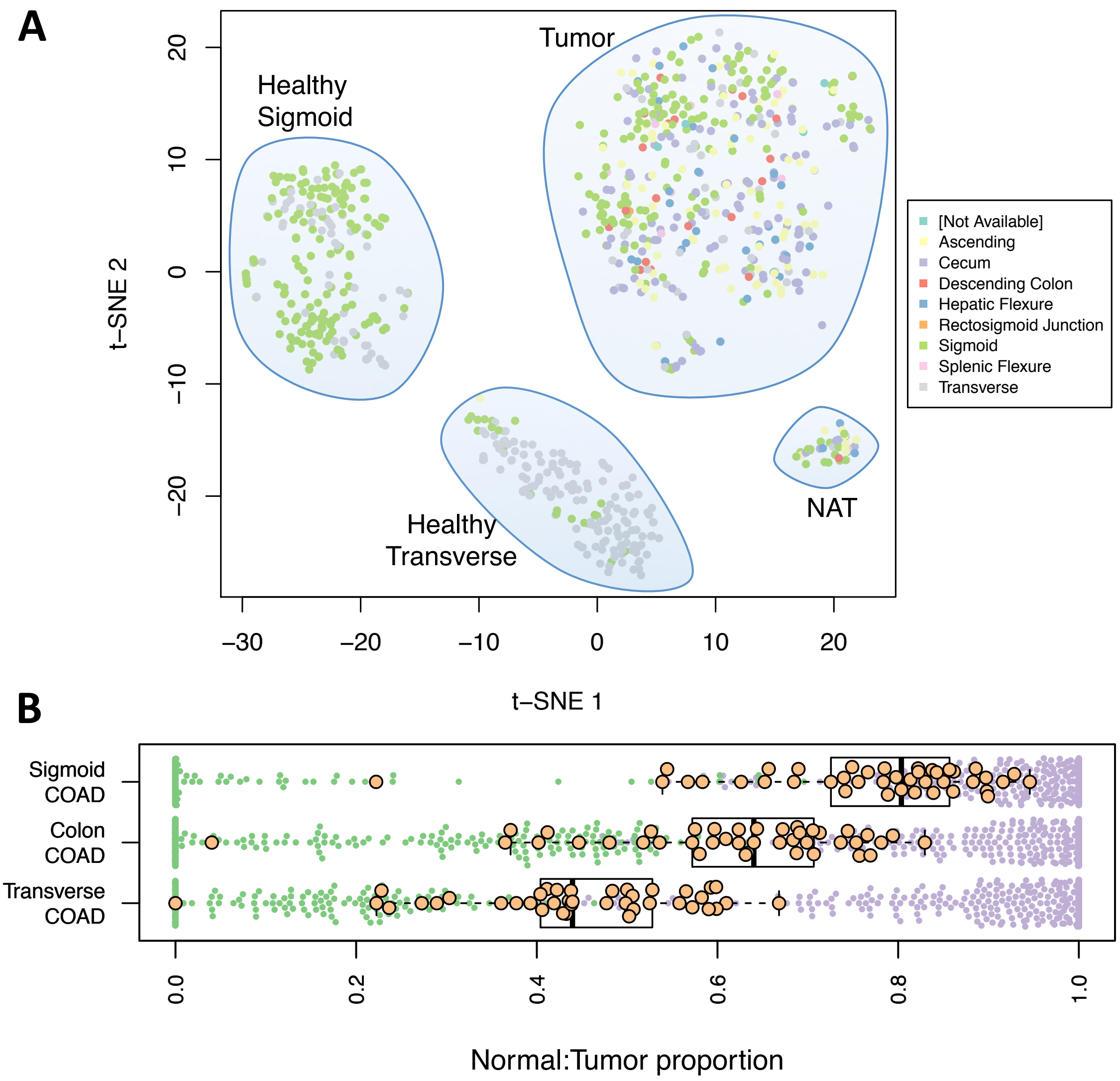

Supplementary Figure 8. Sigmoid and transverse colon clustering. A. t-SNE plot of the colon samples. Instead of three clusters, healthy NAT and tumor, in colon we observed four distinct clusters – the healthy samples from the sigmoid section of the colon cluster away from the transverse samples. In both adjacent and tumor there is no distinction between the colon subdivisions. It is not clear to us what is driving this strong divergent between transverse and sigmoid colon. **B.** Same as figure 2B. Deconvolution analysis of the NAT samples using median expression levels of healthy and tumor as references. The result of the analysis is the fraction of similarity of each NAT sample to the tumor. The small points, normal (green) and tumor (purple) deconvolution fractions, are shown as reference. Removing the healthy sigmoid samples changes the NAT samples to have a median of 0.439, a little less than uterus/ UCEC.

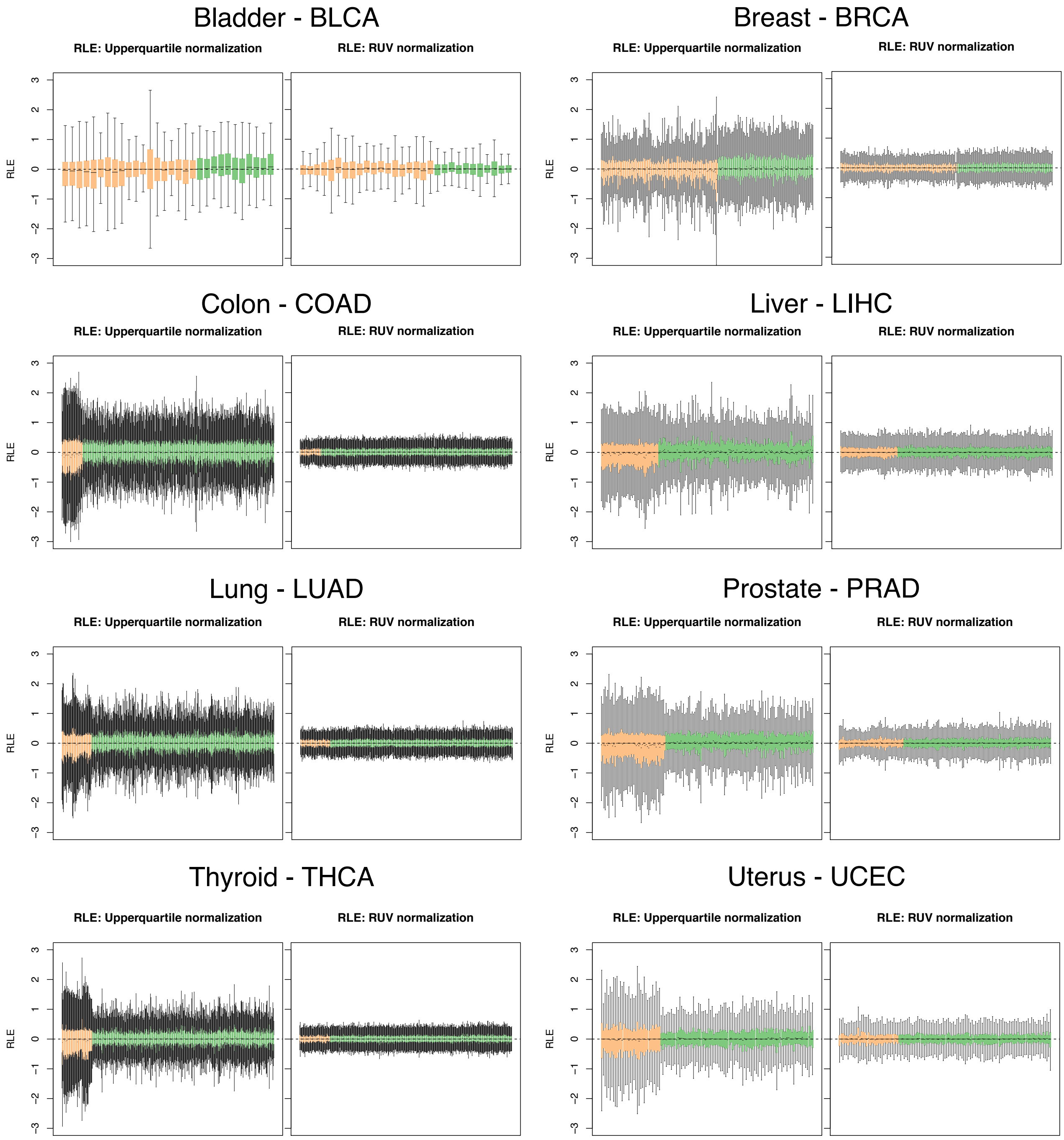

Supplementary Figure 9. **Stringent removal of variation using RUVseq.** The figures show the relative log expression(RLE) using upperquartile and RUVseq normaliztion using housekeeping genes. Without RUV normalization there are differences in RLE between TCGA adjacent normal samples (orange) and GTEx healthy samples (green), after RUV the RLE is less than 1 and no apparent differences are observed.

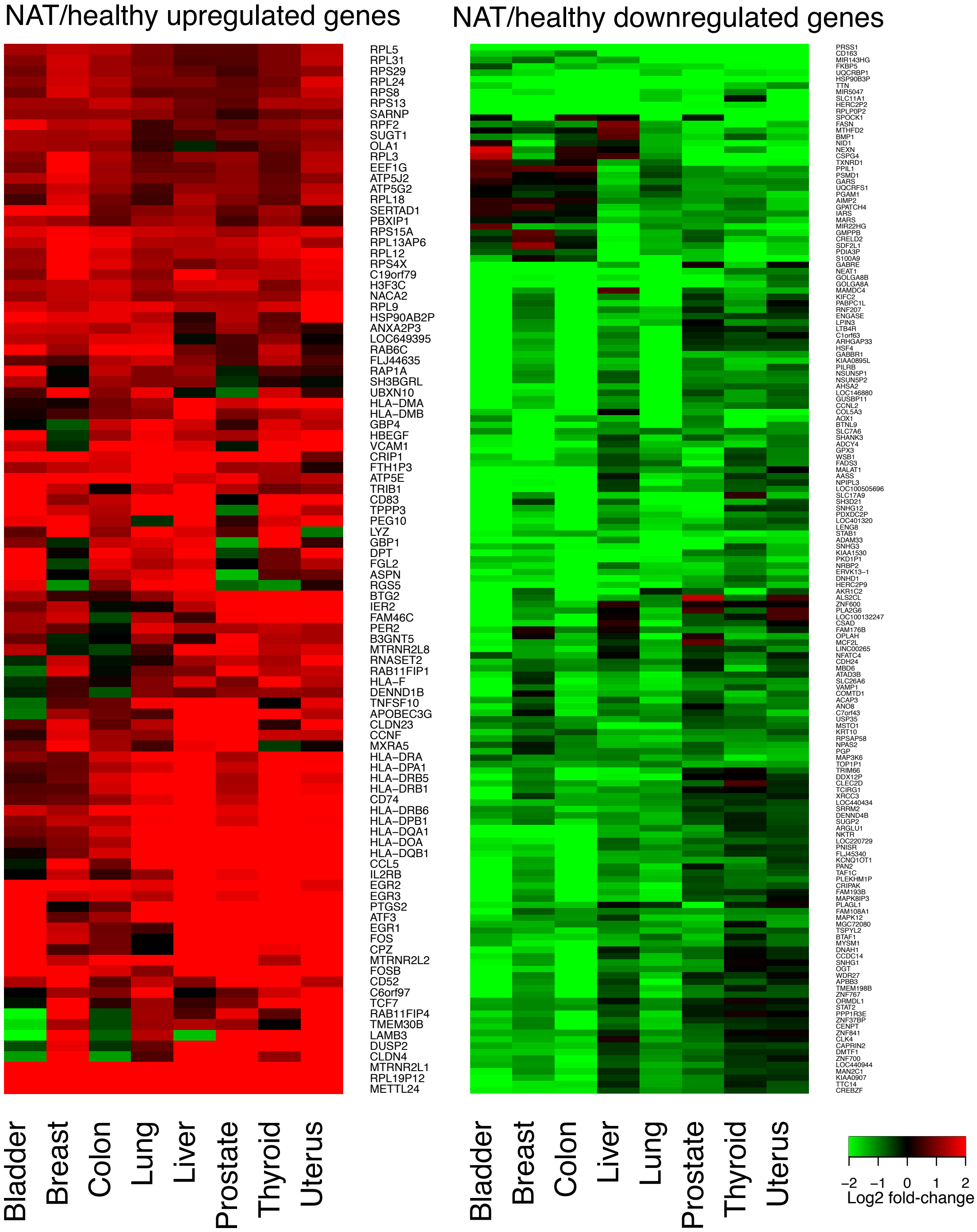

Supplementary Figure 10. Shared differentially expressed genes between NAT and healthy. Fold change)log2) between NAT and healthy for the 98 upregulated genes (right) and 164 downregulated genes (left) that are shared across at least 4 tissue types. 61.2% of the upregulated genes and 73.2% of the downregulated genes are shared across all 8 tissue types.

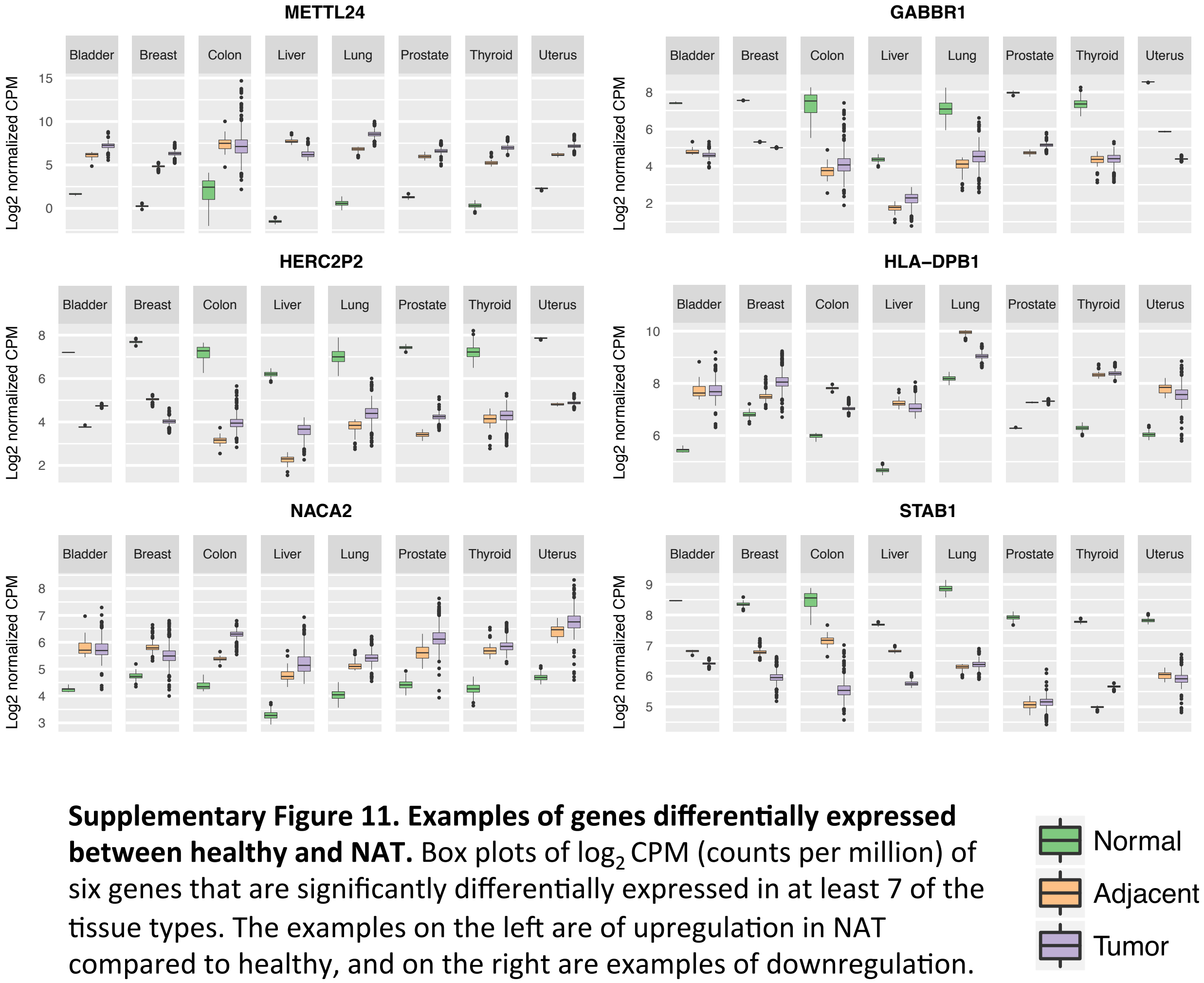

Supplementary Figure 11. Examples of genes differentially expressed between healthy and NAT. Box plots of log_2 CPM (counts per million) of six genes that are significantly differentially expressed in at least 7 of the tissue types. The examples on the left are of upregulation in NAT compared to healthy, and on the right are examples of downregulation. The tumor expression is shown as reference.

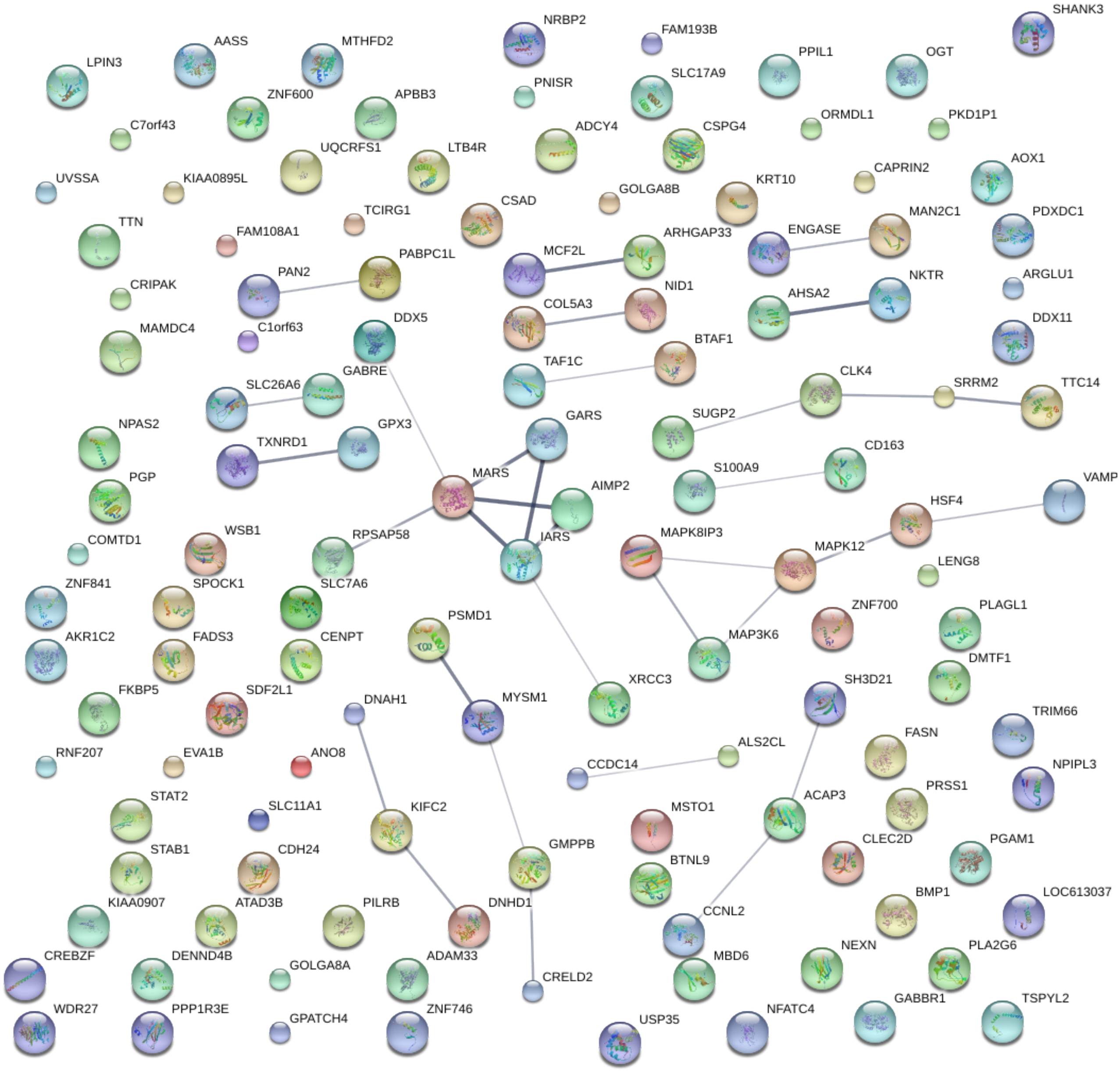

Supplementary Figure 12. Protein-protein interactions of shared downregulated genes in NAT compared to healthy. STRING analysis of the protein-protein interactions of the 164-shared downregulated genes in NAT compared to healthy. The figures shows the interactions between the 132 corresponding proteins, 33 edges are found compared to an expected of 29 (PPI enrichment p-value = 0.229). This network is in striking contrast to the network formed for the upregulated genes.

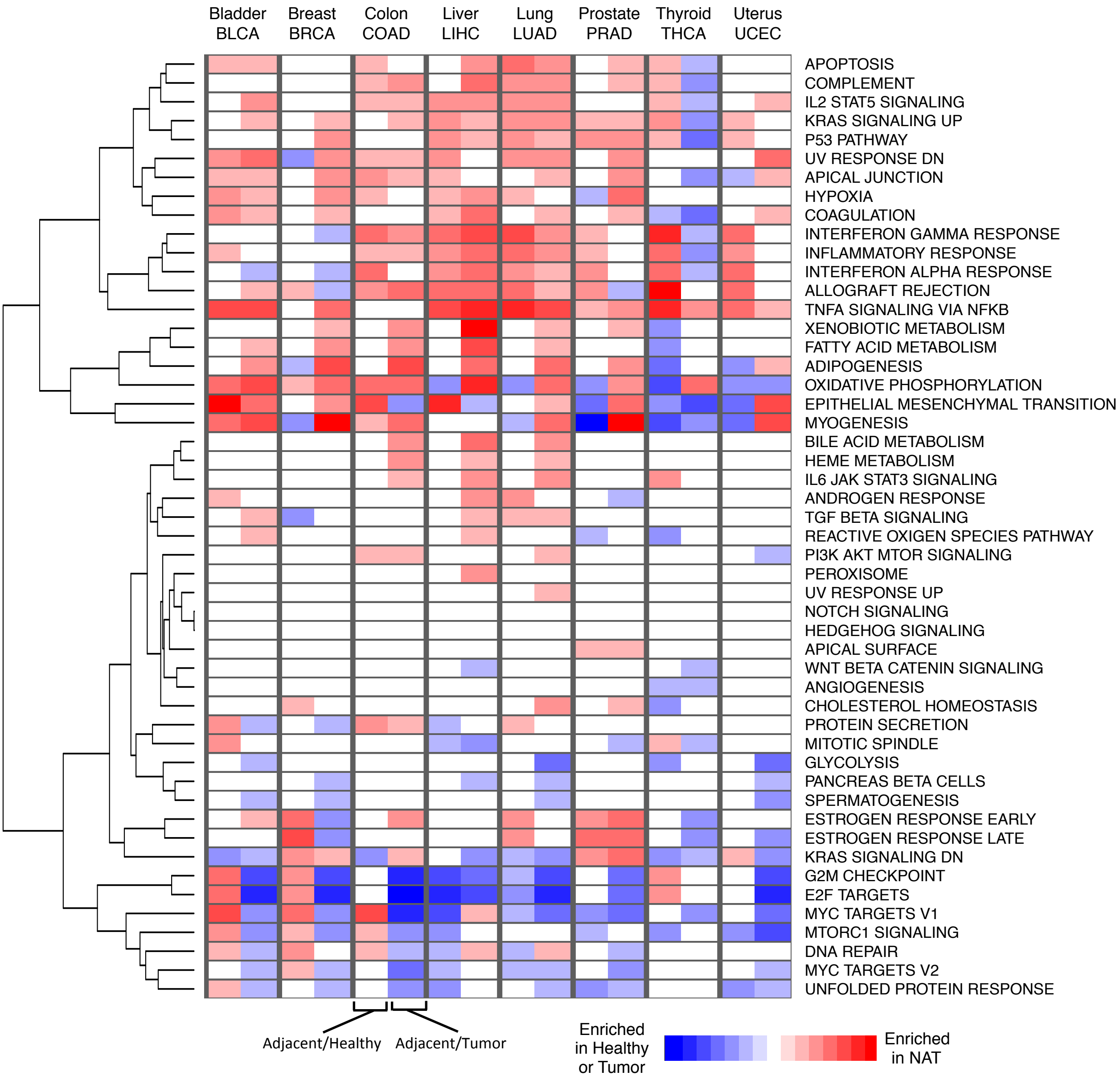

Supplementary Figure 13. Gene set enrichment analysis of the hallmark gene sets. Preranked GSEA was performed for both NAT vs. healthy and NAT vs. tumor differential expression profiles using the 50 hallmark gene sets. The heatmap corresponds to figure 3D, now with the addition of NAT vs. tumor. Averages of this analysis are presented in figure 4B and the full data is in supplementary data 3. The color of each cell is the normalized enrichment scores (NES) (red – positive NES, blue – negative NES). Non-significant comparisons (FDR<1%) are in white. The rows were clustered using complete linkage method.

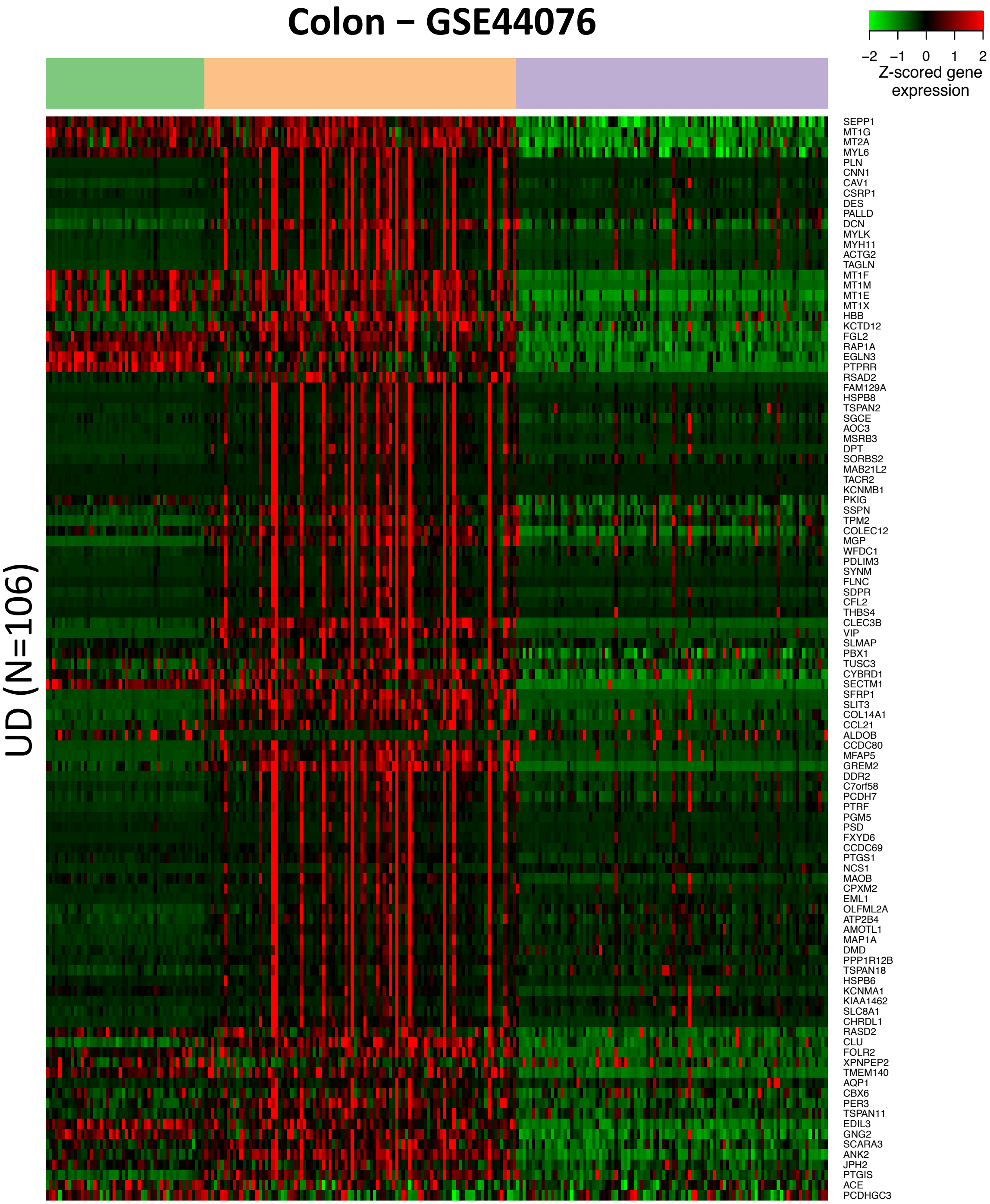

Supplementary Figure 14. Validation of NAT-specific genes in public datasets - Colon. Gene expression profiles in the colon validation cohort of the colon ‘UD’ (NAT-specific activation model) genes found in the TCGA-GTEx data. Of the 106 ‘UD’ genes 92 (86.8%) show a ‘UD’ pattern in the validation cohort as well. The expected number of genes in a null hypothesis is 18.76 (17.7%).

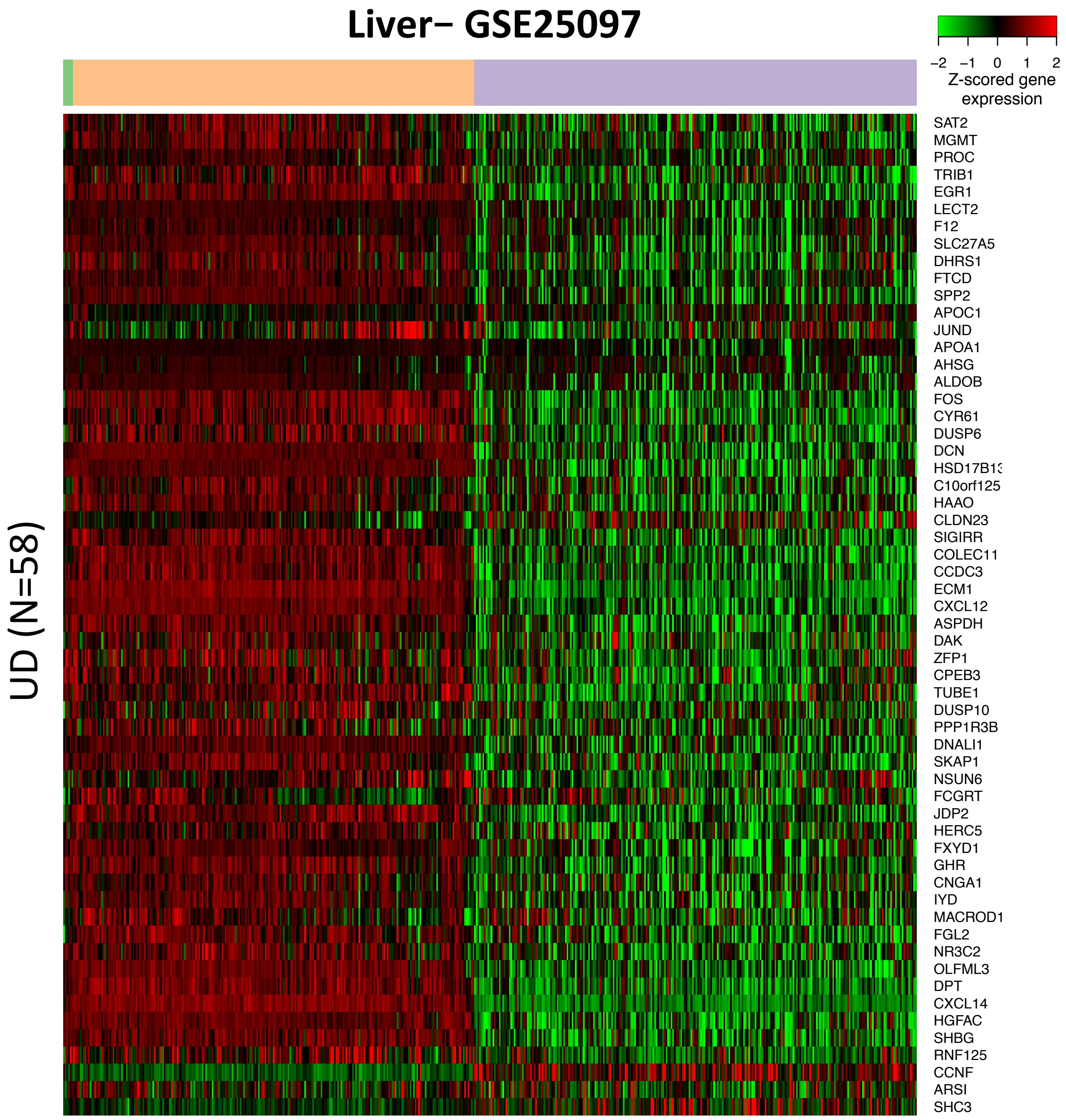

Supplementary Figure 15. Validation of NAT-specific genes in public datasets - Liver. Gene expression profiles in the liver validation cohort of the liver ‘UD’ (NAT-specific activation model) genes found in the TCGA-GTEx data. Of the 58 ‘UD’ genes 46 (79.2%) show a ‘UD’ pattern in the validation cohort as well. The expected number of genes in a null hypothesis is 15.66 (27.0%).

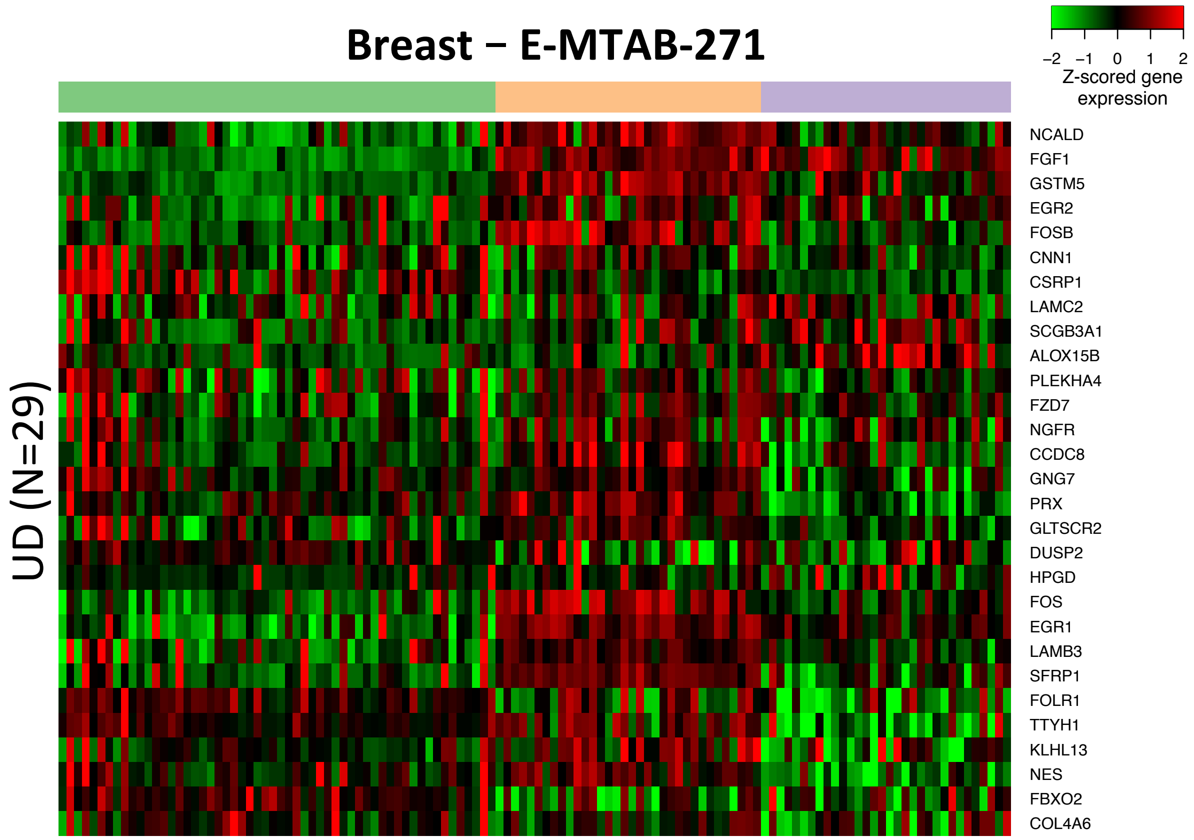

Supplementary Figure 16. Validation of NAT-specific genes in public datasets - Breast. Gene expression profiles in the breast validation cohort of the breast 'UD' (NAT-specific activation model) genes found in the TCGA-GTEx data. Of the 29 'UD' genes 20 (69.0%) show a 'UD' pattern in the validation cohort as well. The expected number of genes in a null hypothesis is 9.89 (34.1%).

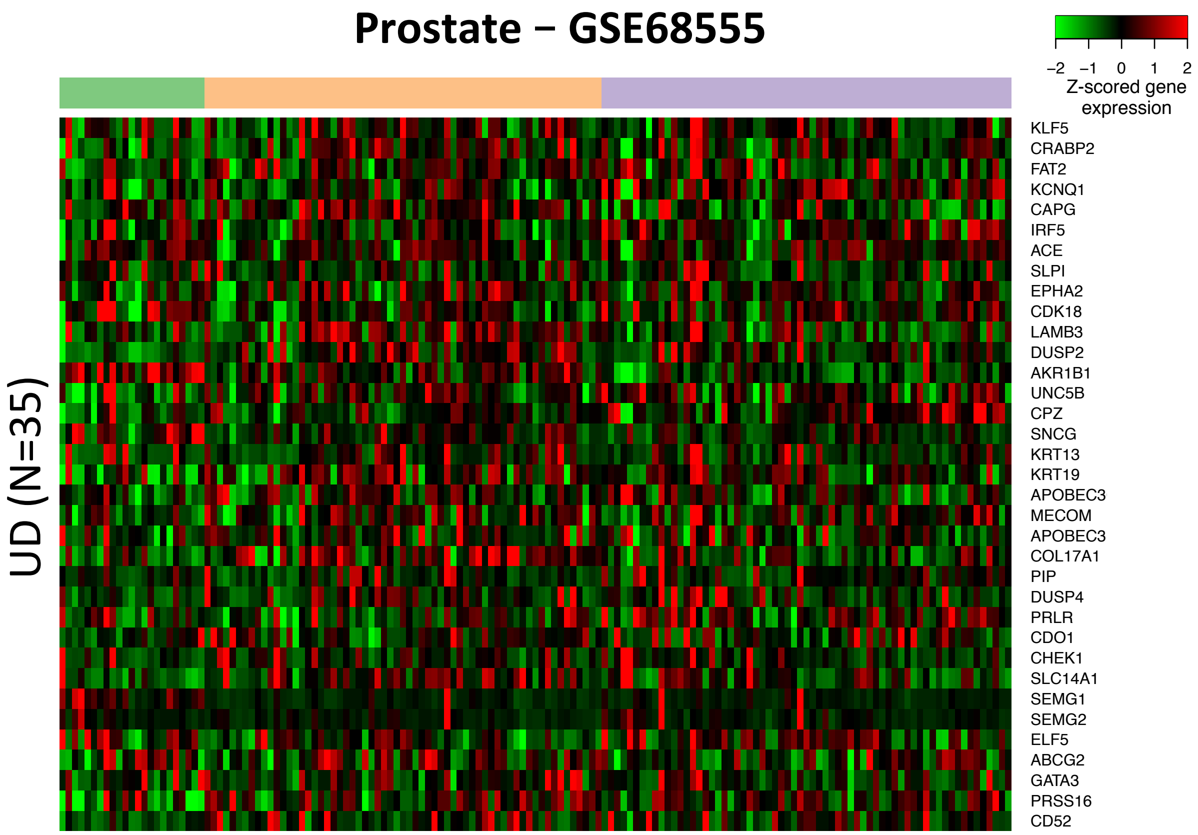

Supplementary Figure 17. Validation of NAT-specific genes in public datasets - Prostate. Gene expression profiles in the prostate validation cohort of the prostate 'UD' (NAT-specific activation model) genes found in the TCGA-GTEx data. Of the 35 'UD' genes 17 (48.4%) show a 'UD' pattern in the validation cohort as well. The expected number of genes in a null hypothesis is 6.58 (18.8%).

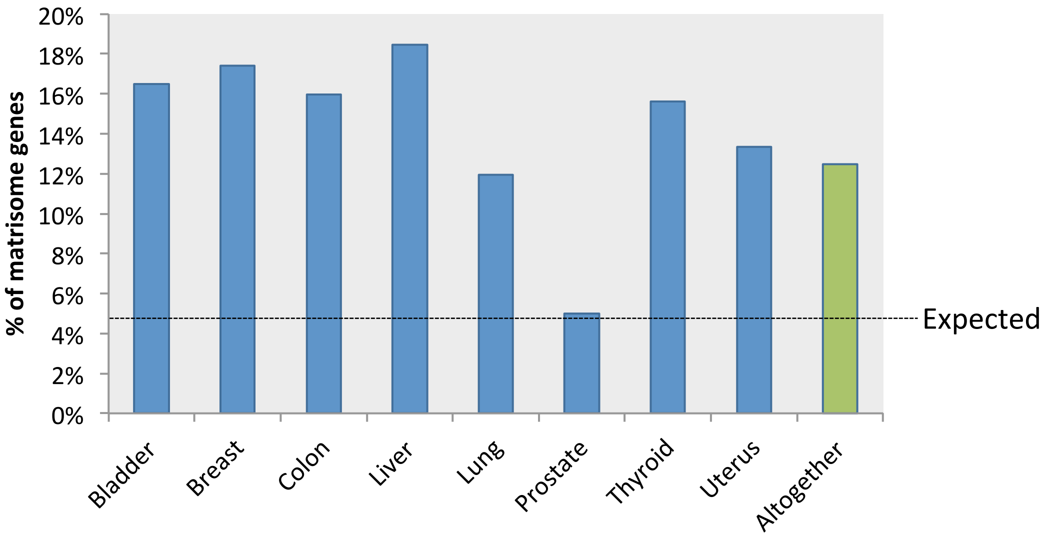

Supplementary Figure 18. Upregulated NAT specific genes enriched with extracellular matrix gene. We bars show the percentage of genes of upregulated in NAT compared to healthy and downregulated compared to tumor that are part of the extracellular matrix (ECM). We use here the list of 1040 ECM genes generated by Naba et al. (Matrix Biology, 2016). In 7 of the tissue types we observed significant enrichments (FDR<1%) compared to the expected by random.

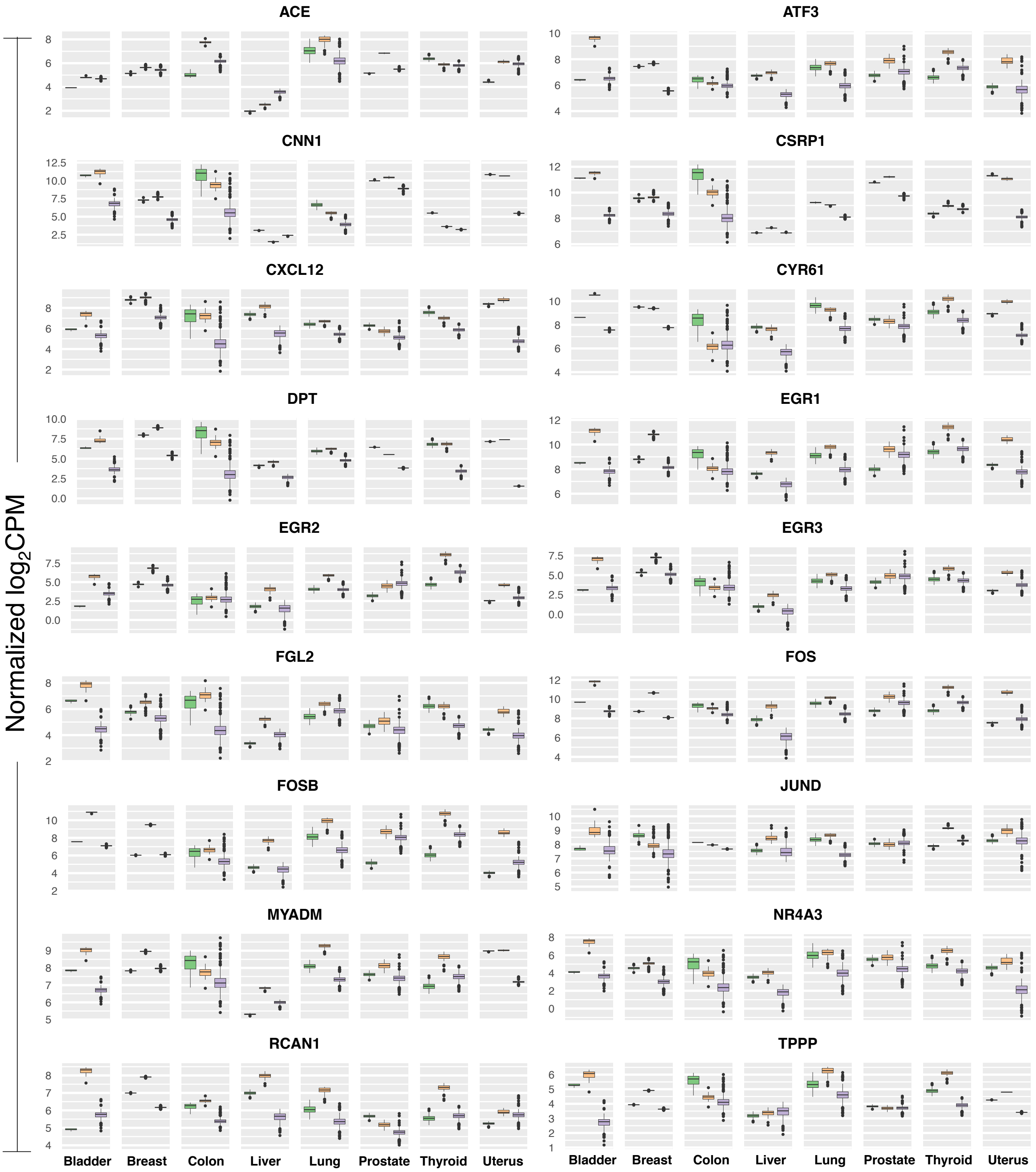

Supplementary Figure 19. Expression profiles of the 18-shared TASA genes. Boxplots of the normalized $\log_2$ CPM of the 18 genes that are specifically upregulated in NAT in at least 3 tissue types.

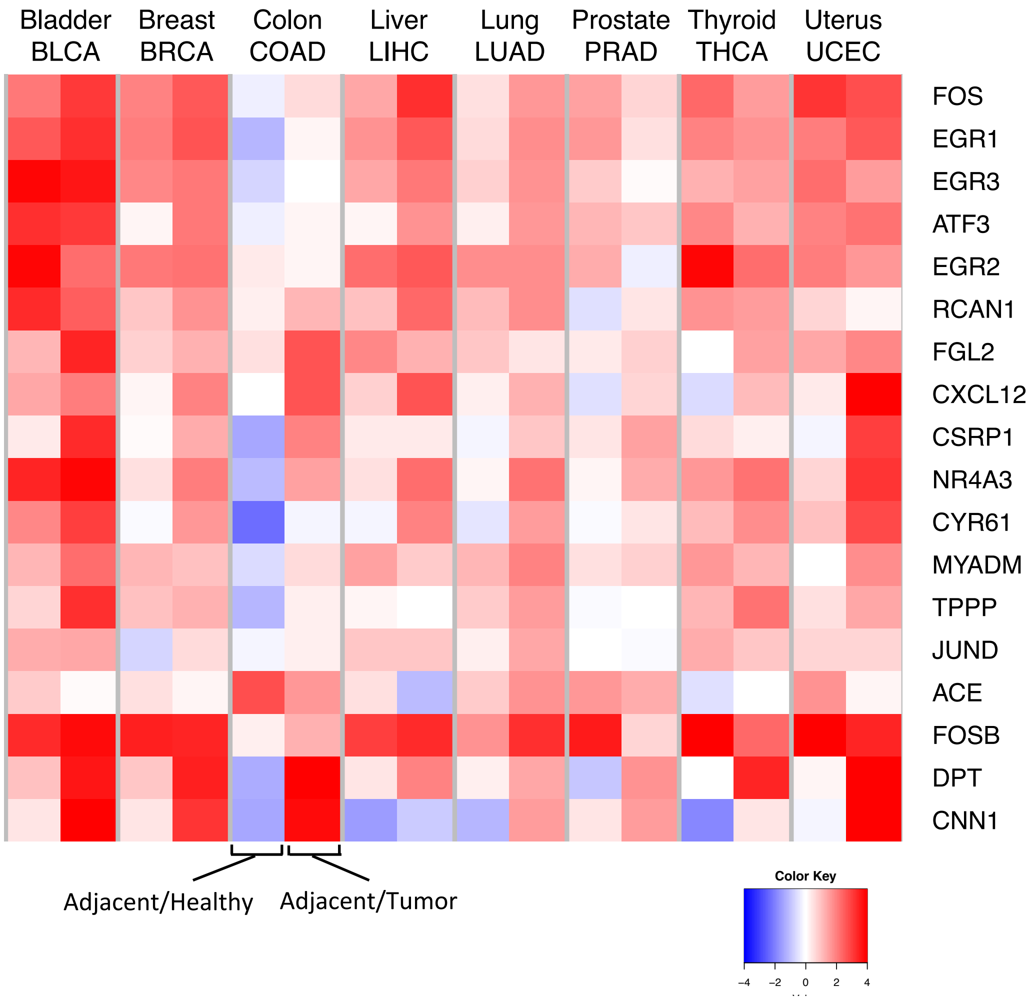

Supplementary Figure 20. Average expression profiles of the 18-shared TASA genes. Log fold-change differences between NAT and healthy and NAT and tumor across tissue sites. 85.8% of the comparisons across the 18 shared-NAT specific genes are positive (higher levels in NAT compared to healthy or tumor).

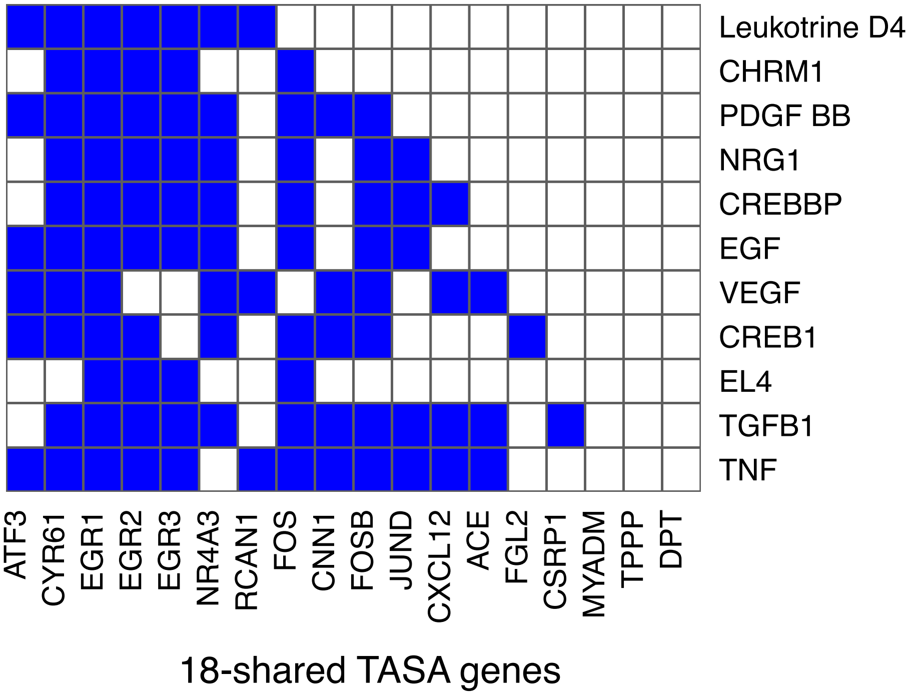

Supplementary Figure 21. **Upstream regulators analysis of the 18 shared-TASA genes.** Using the Ingenuity Pathway Analysis software we search for significant upstream regulators of the 18-shared TASA genes. Several regulators can induce the shared TASA genes network, with a strong preference for blood vessels-related regulators such as leukotriene D4, PDGF-BB and VEGF.

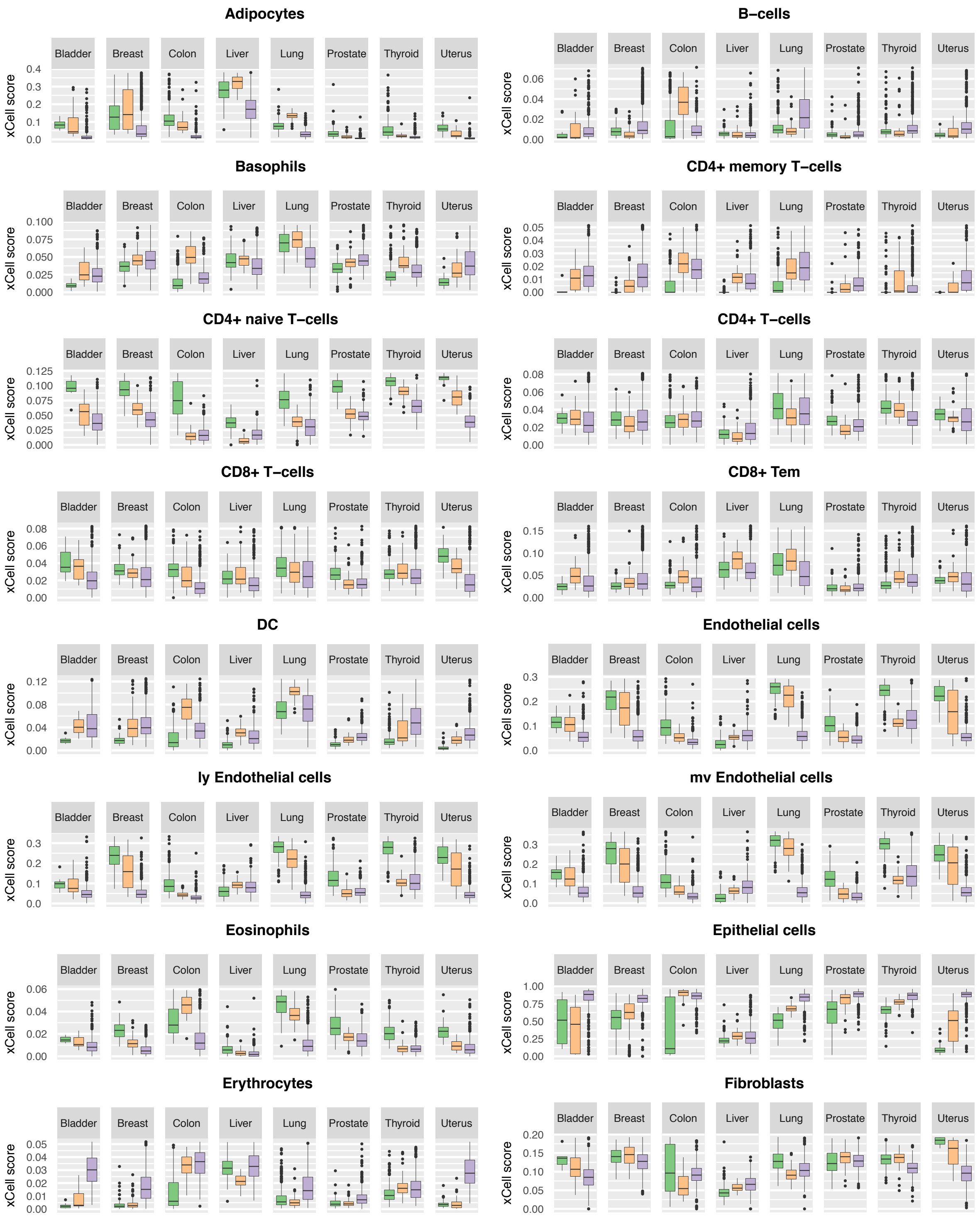

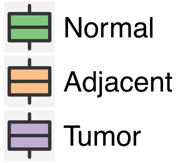

Supplementary Figure 22. Tissue composition of enrichment scores (1). Boxplots presenting the xCell scores of cell types analyzed for all samples, divided to tissue types and conditions. The y axis was truncated to present scores between 0 and the 95% percentile of all samples.

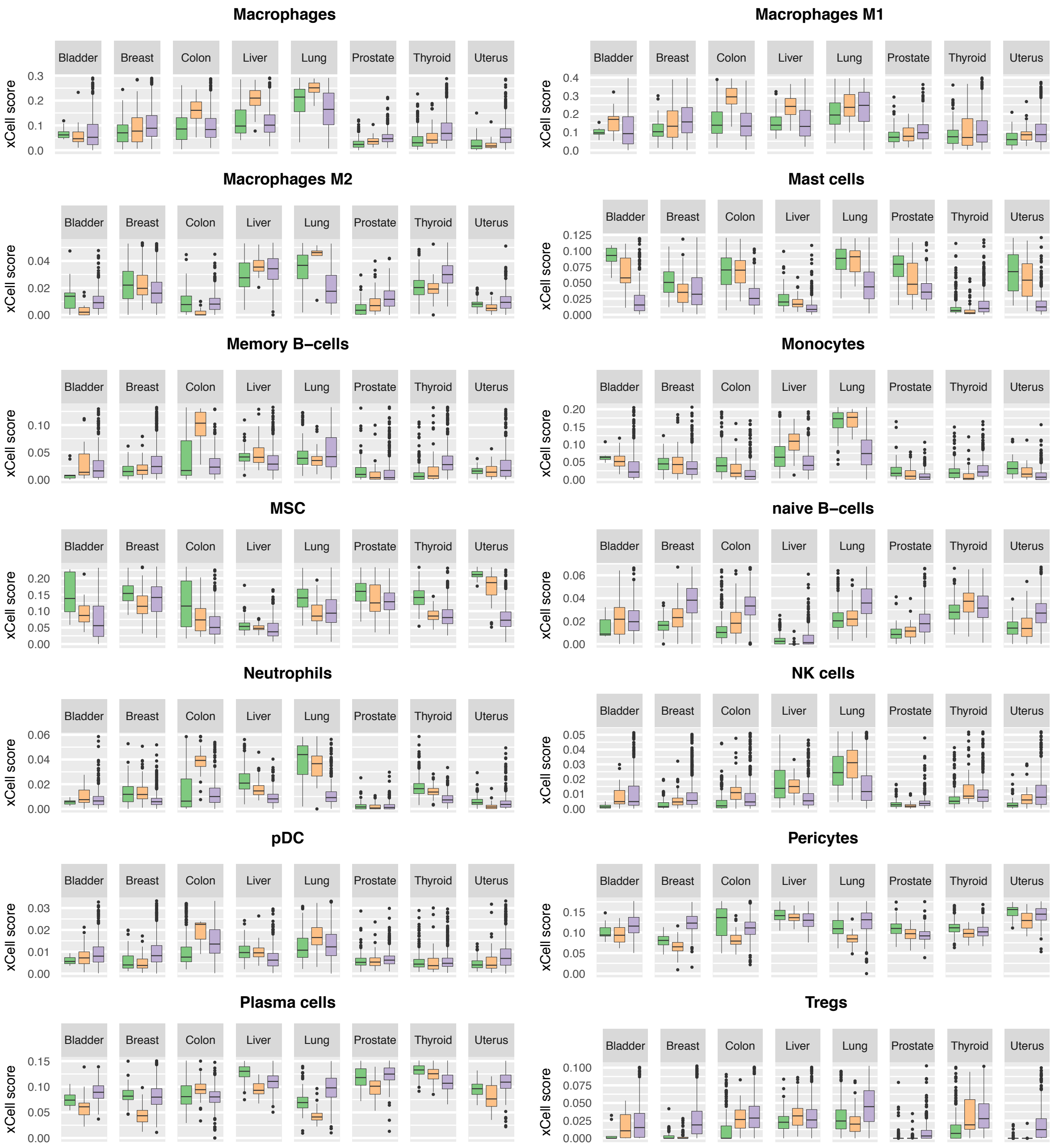

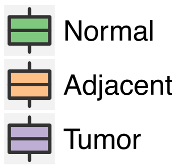

Supplementary Figure 23. Tissue composition of enrichment scores (2). Boxplots presenting the xCell scores of cell types analyzed for all samples, divided to tissue types and conditions. The y axis was truncated to present scores between 0 and the 95% percentile of all samples.

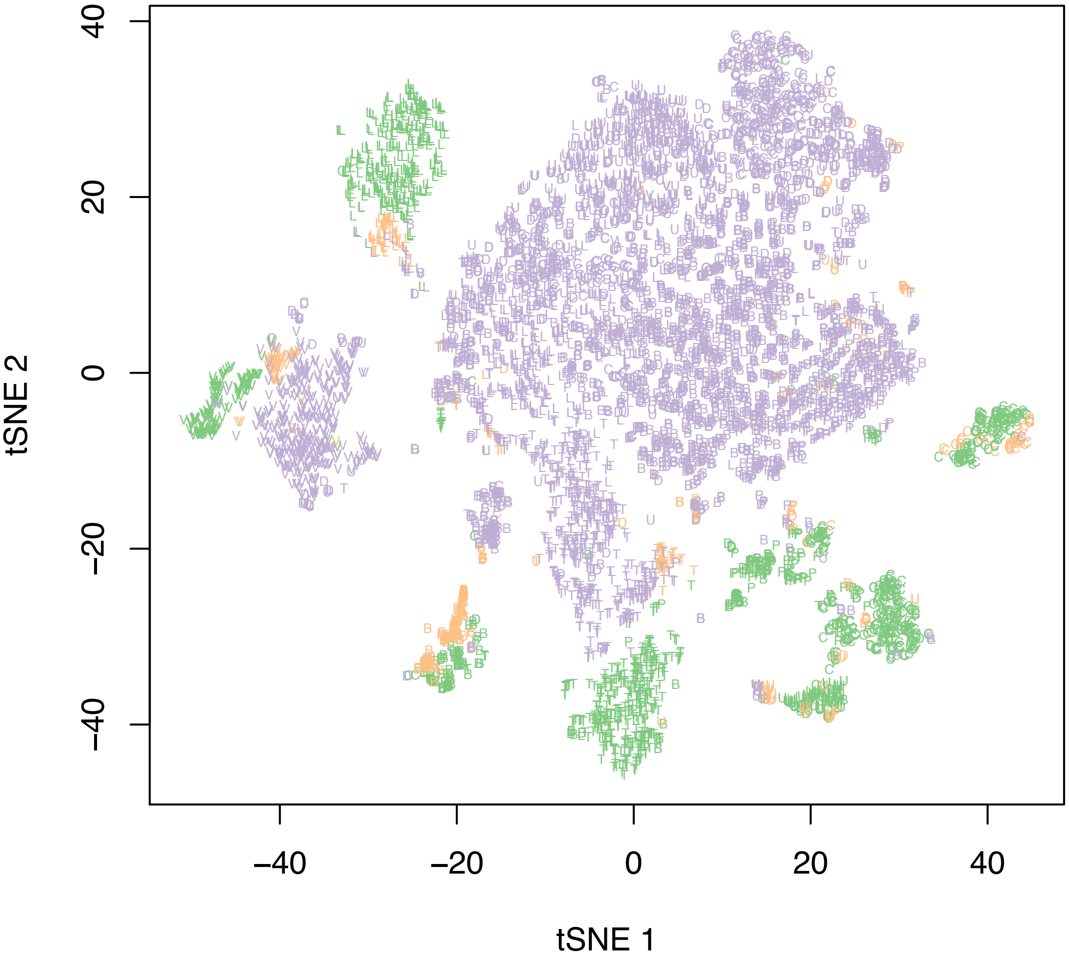

Supplementary Figure 24. Tissue composition of NAT resembles more closely the healthy tissue than the tumor. A t-SNE plot of all 6,486 samples according to their xCell scores of 30 cell types. Colors represent the condition – purple – tumor, orange – NAT, green – healthy. Letters represent the tissue type: L – lung, B – breast, C – colon, D – bladder, V – liver, T – thyroid, P – prostate, U – uterus. Tumor samples are clustered together, irrespective of the tissue type, while healthy and NAT samples of each tissue type are clustered together according to the tissue type. Liver samples form of all conditions form a distinct cluster. Thyroid NATs are clustered with the tumors.

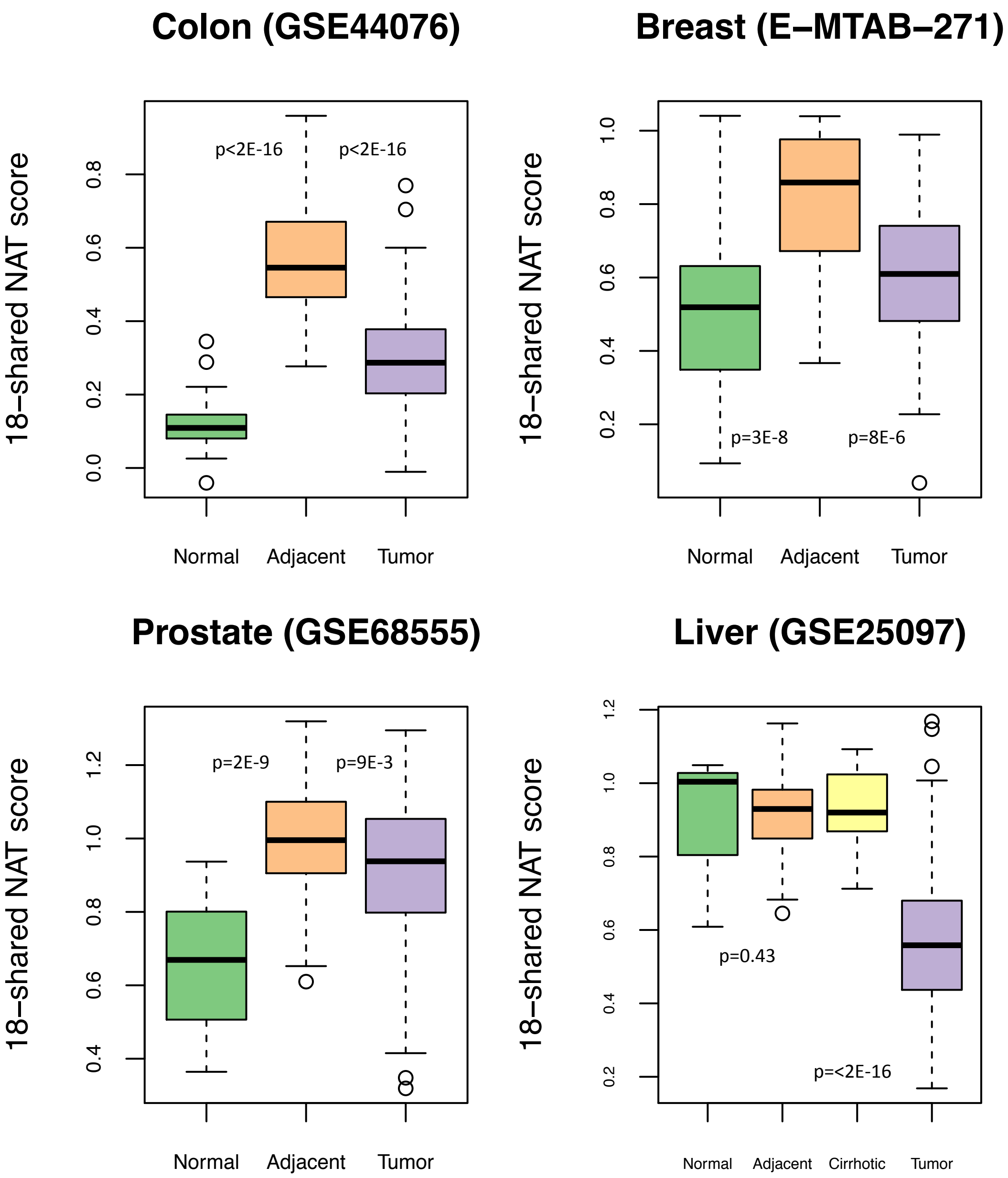

Supplementary Figure 25. The TASA signature in independent microarray datasets. Boxplots of the ssGSEA scores for the 18-shared TASA genes in 4 microarray datasets. The TASA score is enriched in NAT samples in 3 of the cohorts. P-values from a Wilcoxon rank-sum test are presented between normal/adjacent and adjacent/tumor. In liver the normal samples do not show a reduced score, possibly because the sample size is low (6 samples).

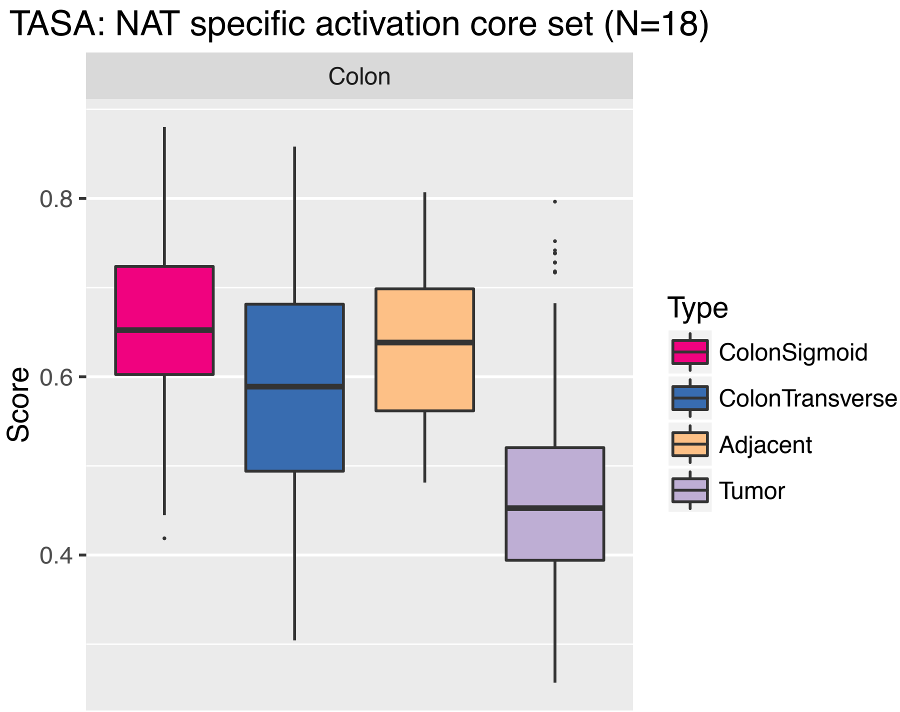

Supplementary Figure 26. The TASA signature in sigmoid and transverse colon. Boxplots of the ssGSEA scores of the colon samples. The GTEx samples from the sigmoid colon have higher levels of TASA score than NAT, however the transverse colon samples show lower levels than NAT in agreement with other tissue types.

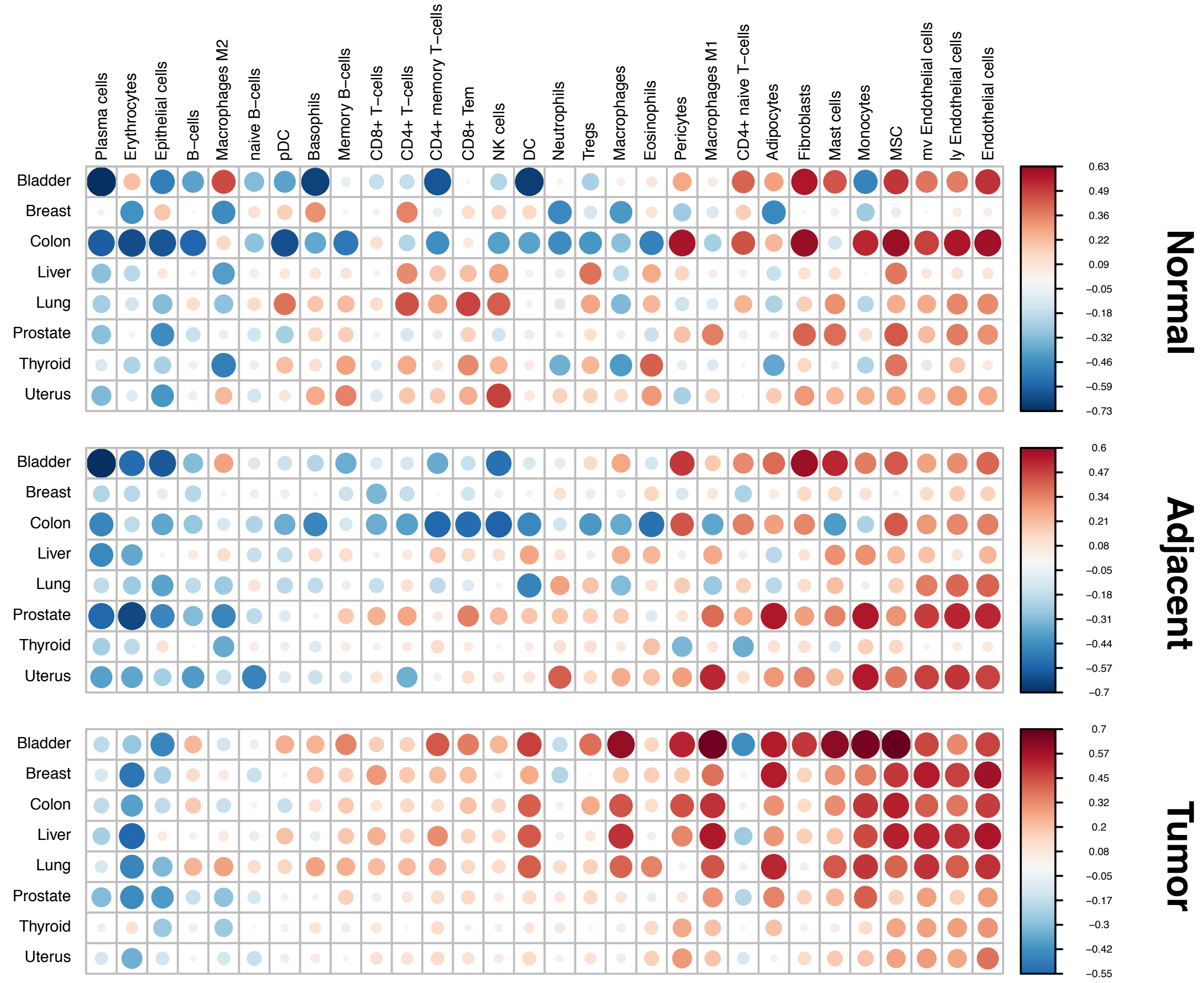

Supplementary Figure 27. Cell types correlation with TASA score. Spearman correlation of the cell types xCell scores with the TASA shared TASA ssGSEA scores. Many differences are observed between tissue types. Nevertheless, the top correlated cell types are correlated with TASA in most tissue types.

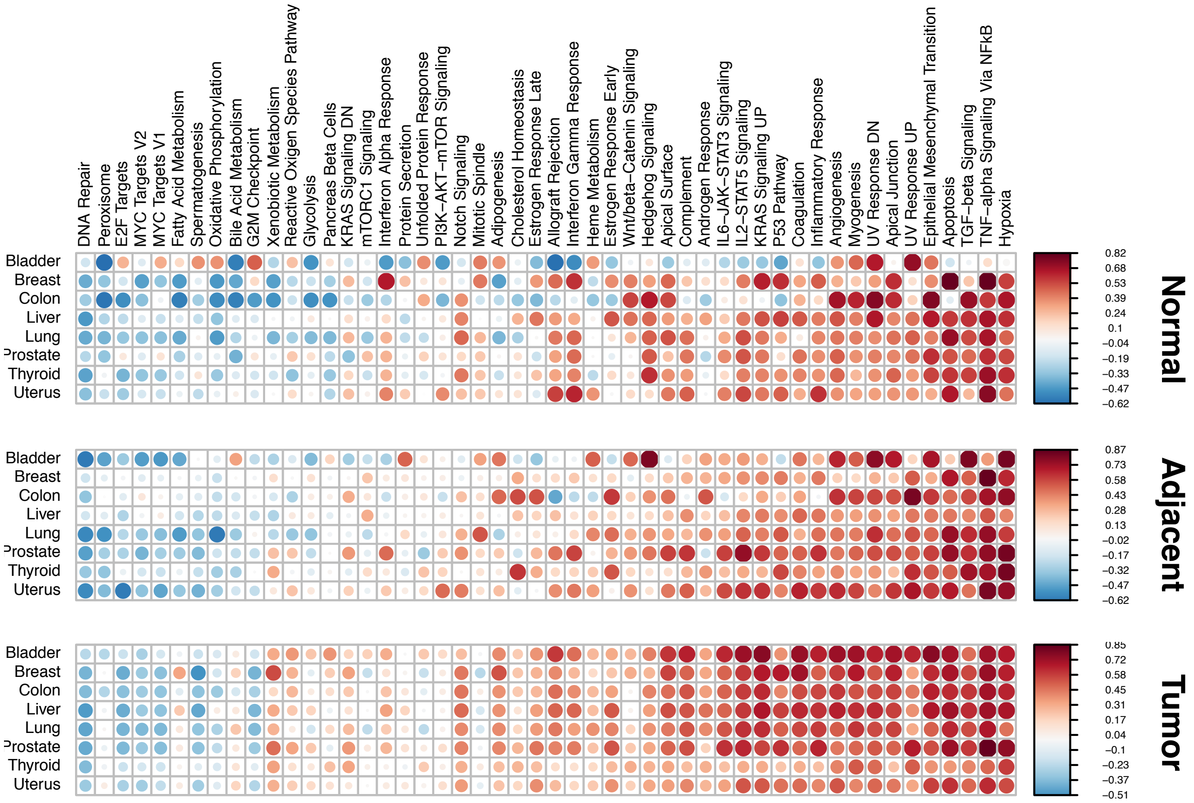

Supplementary Figure 28. Hallmark gene sets correlation with TASA score. Spearman correlation of the hallmark gene sets ssGSEA scores with the TASA shared TASA ssGSEA. High concordance between tissue types and conditions is observed.

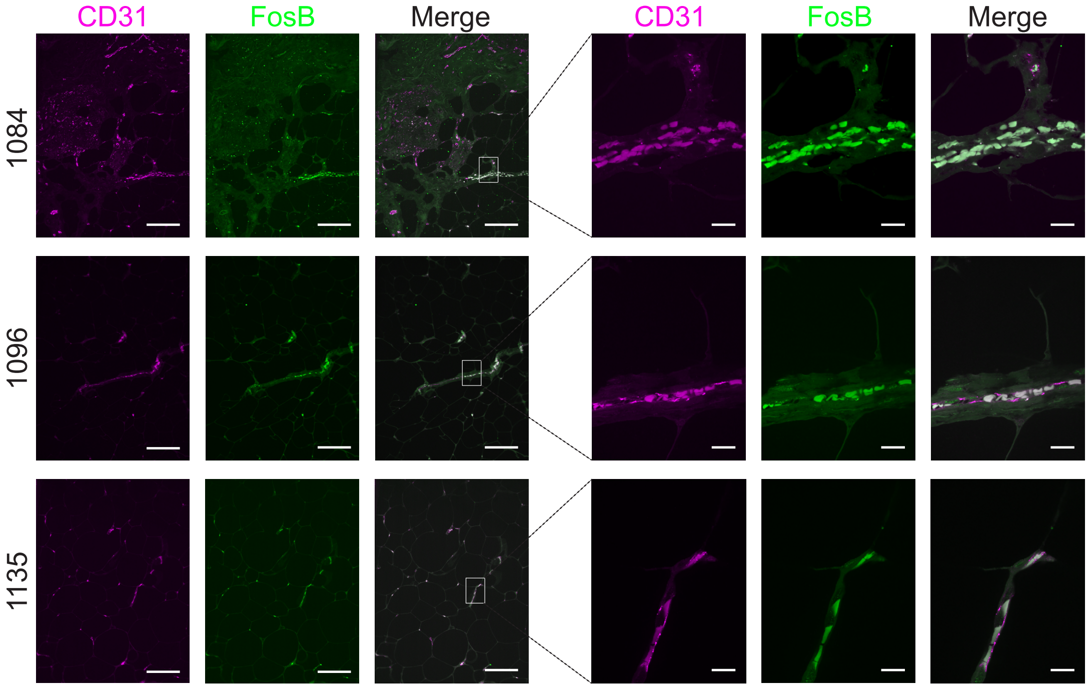

Supplementary Figure 29. Colocalization of FOSB to endothelial cells in NAT. Immunofluorescent staining for CD31, an endothelial cell marker, and FosB protein in NAT of three human breast tumor excision specimens. Remarkably, in all three specimens both markers are highly colocalized (Costes p-value < 1e-6).

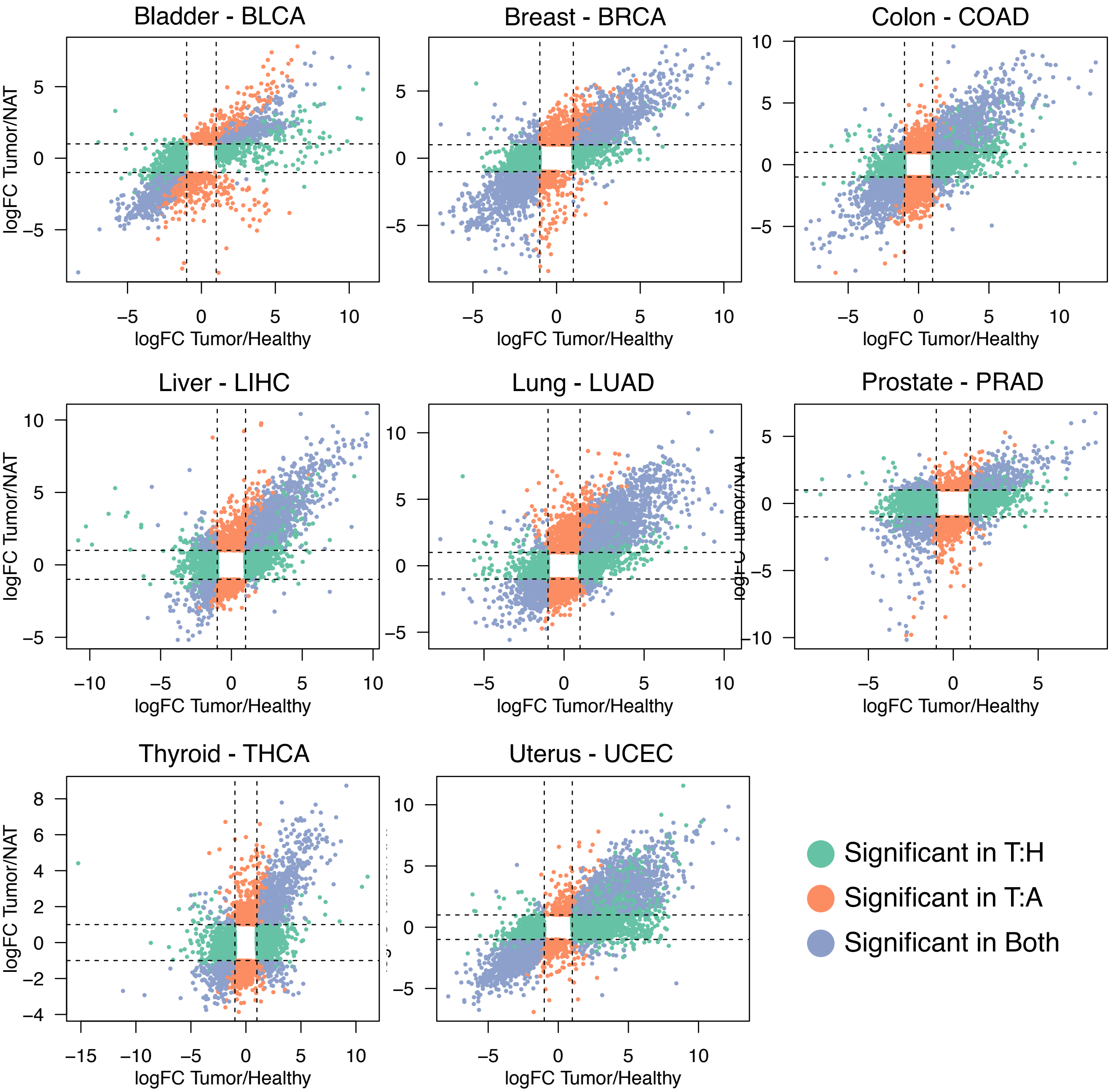

Supplementary Figure 30. Differential expression analyses using healthy or NAT as controls. Scatter plots of the fold changes from differential expression analysis of tumor vs. normal samples. In the x-axis the normal samples are the GTEx healthy samples, and in the y-axis the normal samples are TCGA NAT samples. The green dots are genes significant (p-value<0.05 Bonferroni corrected, Fold-change>1) only in the Tumor/Healthy, the orange are significant only in Tumor/NAT and blue are significant in both.

Supplementary Table 2:
Summary counts of differentially expressed genes between healthy, NAT and tumor samples

Table S1. Summary counts of differentially expressed genes between healthy, NAT and tumor samples. Number of differentially expressed (DE) genes (Bonferroni corrected p-value<0.05, 2-fold expression change, and $log_2CPM>3$) in each tissue type between NAT and healthy (columns 1 and 3) and tumor and adjacent (columns 2 and 4). In the bottom we present the number of genes that are shared between more than 3,4 and 5 tissue types. In parenthesis – the number of genes expected by random (based on 1000 randomly shuffling the DE genes). A significant portion of the genes are shared among tissue types. For example 98 genes are found to higher expression in at least 4 of the tissue types in NAT compared to healthy. In a null hypothesis the expected number of shared genes in at least 4 tissue types is only 1.2.

	**# of upregulated genes**		**# of downregulated genes**	
	**NAT > Healthy**	**Tumor > NAT**	**NAT < Healthy**	**Tumor < NAT**
**Bladder**	244	261	486	642
**Breast**	1038	612	711	842
**Colon**	331	739	913	933
**Liver**	328	379	477	560
**Lung**	467	671	747	790
**Prostate**	285	182	315	500
**Thyroid**	443	480	474	329
**Uterine**	378	668	306	1005
**Average**	439.25	499	553.625	700.125
**Shared (N>=3)**	223 (27.4)	369 (41.6)	420 (56.0)	607 (110.4)
**Shared (N>=4)**	98 (1.2)	163 (2.3)	164 (3.4)	332 (8.7)
**Shared (N>=5)**	45 (0.3)	65 (0.8)	72 (0.9)	154 (0.4)

Supplementary Table 2:
Summary counts of differentially expressed genes grouped to expression models

Table S2. Summary counts of differentially expressed genes grouped to expression models. Differentially expressed genes (DEGs) were divided to nine expression models: each gene can be upregulated (U), downregulated (D) or not differentially expressed (stable, S) in NAT vs. healthy (A:H) and Tumor vs. NAT (T:A). The table shows the number of DEGs in each expression model across tissue types. The null model (SS) is not presented.

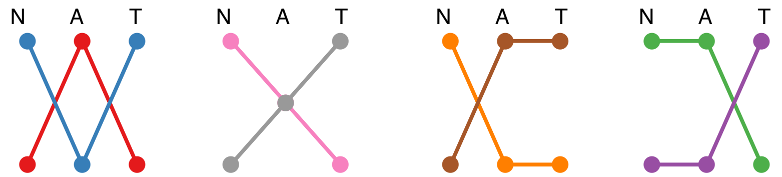

	NAT-specific		Gradient		Tumor-like		Normal-like	
	**UD**	**DU**	**UU**	**DD**	**US**	**DS**	**SU**	**SD**
**Bladder/BLCA**	97	31	1	2	111	397	167	330
**Breast/BRCA**	46	15	187	164	376	274	299	474
**Colon/COAD**	119	125	19	119	147	390	398	408
**Liver/LIHC**	65	81	5	21	212	214	225	344
**Lung/LUAD**	159	177	25	46	192	305	280	348
**Prostate/PRAD**	60	8	3	31	144	154	123	234
**Thyroid/THCA**	64	73	33	32	254	263	317	200
**Uterine/UCEC**	45	41	45	62	210	135	452	721
**Average**	81.9	68.9	39.8	59.6	205.7	266.0	282.6	382.3

Supplementary Table 3:
Upstream regulators of the NAT specific genes

Table S3. Upstream regulators of the NAT specific genes. Using Ingenuity Pathway Analysis © we performed an upstream regulators analysis, which finds regulators that are known to be upstream of the input genes(by manual curating published finding). The table shows the top significant upstream regulators using the 18-shared NAT specific genes as input. In addition, we performed the analysis with the TASA genes of each tissue type as input, and p-values are shown. The last column shows the number of tissue types that were the regulator was found highly significant (*p-value* < 1e-5). All the top upstream regulators are shared among at least 4 tissue types.

Upstream Regulator	Molecule Type	P-value of enrichment									# <1e-5
		Shared NAT	Bladder	Breast	Colon	Liver	Lung	Prostate	Thyroid	Uterus	
**leukotriene D4**	chemical - endogenous	9.00E-15	5.52E-18	5.37E-03	1.00E+00	9.88E-03	3.32E-09	1.00E+00	2.37E-16	7.76E-10	4
**CHRM1**	g-protein coupled receptor	1.22E-13	1.40E-07	1.60E-06	1.00E+00	4.16E-06	2.85E-03	1.00E+00	2.63E-13	5.06E-09	5
**PDGF BB**	complex	6.82E-13	1.60E-19	4.11E-04	2.36E-02	2.48E-05	1.78E-06	1.00E+00	2.03E-19	3.87E-13	4
**formaldehyde**	chemical - endogenous	1.51E-11	5.50E-06	2.36E-09	1.00E+00	9.73E-07	1.52E-02	1.00E+00	1.04E-10	2.05E-07	5
**EGF**	growth factor	2.93E-11	1.46E-08	3.74E-04	3.78E-02	6.19E-06	2.58E-05	3.68E-02	2.96E-11	5.22E-11	4
**Vegf**	group	3.39E-11	2.24E-10	1.63E-02	9.54E-04	5.48E-05	6.56E-09	1.00E+00	6.17E-06	1.76E-08	4
**CREB1**	transcription regulator	6.57E-11	5.68E-09	6.04E-04	1.00E+00	1.46E-02	3.21E-06	1.00E+00	9.81E-11	4.58E-07	4
**ELK4**	transcription regulator	1.26E-10	1.40E-07	1.60E-06	1.00E+00	4.73E-04	2.85E-03	1.00E+00	9.15E-11	5.06E-09	4
**TGFB1**	growth factor	1.75E-10	1.27E-16	5.77E-05	6.52E-13	1.20E-02	1.77E-14	2.81E-03	6.06E-10	2.77E-05	4
**TNF**	cytokine	1.95E-10	3.96E-13	2.15E-06	7.97E-05	3.99E-08	4.76E-15	1.38E-05	1.28E-18	1.24E-07	6
**LDL**	complex	2.95E-10	3.03E-10	1.01E-04	1.00E+00	2.63E-02	7.58E-09	2.32E-02	4.69E-14	4.12E-10	4
**leukotriene C4**	chemical - endogenous	3.48E-10	3.39E-09	3.29E-06	1.00E+00	7.49E-04	4.48E-03	1.00E+00	3.45E-10	1.39E-08	4
**beta-estradiol**	chemical - endogenous	4.28E-10	3.88E-13	5.22E-04	6.44E-07	5.67E-05	1.08E-12	4.72E-04	2.43E-09	1.12E-05	4
**Ca2+**	chemical - endogenous	5.22E-10	1.28E-06	1.64E-03	4.18E-02	5.16E-03	2.01E-08	1.00E+00	2.54E-08	7.32E-06	4
**SRF**	transcription regulator	3.00E-09	3.38E-14	2.45E-07	9.76E-07	1.00E+00	3.99E-04	1.00E+00	6.18E-11	1.41E-07	5
