# Supplementary material for: Comprehensive analysis of normal adjacent to tumor transcriptomes
Source: Nat Commun. 2017 Oct 20;8:1077. doi: 10.1038/s41467-017-01027-z (PMC5651823; doi:10.1038/s41467-017-01027-z)
Supplement: Supplementary file 2 — Description of Additional Supplementary Files [file 41467_2017_1027_MOESM2_ESM.pdf]

Description of Additional Supplementary Files

File Name: Supplementary Data 1
Description: Differential expression analysis between healthy tissues and NAT in each of the eight tissue types.

File Name: Supplementary Data 2
Description: Genes upregulated/downregulated in comparison of NAT and healthy, and the number of tissue types they were perturbed in.

File Name: Supplementary Data 3
Description: GSEA hallmark gene sets analysis of NAT vs. healthy tissues and NAT vs. tumor.

File Name: Supplementary Data 4
Description: Differential expression analysis between tumors and NAT in each of the eight tissue types.

File Name: Supplementary Data 5
Description: Top 100 upstream regulators of the 18-shared TASA genes as found by Ingenuity Pathway Analysis.

File Name: Supplementary Data 6
Description: Spearman coefficients of the correlations between TASA in the NAT tissue with the expression of genes in the paired tumor samples in each tumor type.

File Name: Supplementary Data 7
Description: Enrichment scores of 30 cell types generated by xCell.
